# The SWR1 Histone Replacement Complex Causes Genetic Instability and Genome-Wide Transcription Misregulation in the Absence of H2A.Z

**DOI:** 10.1371/journal.pone.0012143

**Published:** 2010-08-12

**Authors:** Macarena Morillo-Huesca, Marta Clemente-Ruiz, Eloísa Andújar, Félix Prado

**Affiliations:** 1 Department of Molecular Biology, CABIMER-CSIC, Seville, Spain; 2 Genomics Unit, CABIMER-CSIC, Seville, Spain; National Cancer Institute, United States of America

## Abstract

The SWR1 complex replaces the canonical histone H2A with the variant H2A.Z (Htz1 in yeast) at specific chromatin regions. This dynamic alteration in nucleosome structure provides a molecular mechanism to regulate transcription, gene silencing, chromosome segregation and DNA repair. Here we show that genetic instability, sensitivity to drugs impairing different cellular processes and genome-wide transcriptional misregulation in *htz1Δ* can be partially or totally suppressed if SWR1 is not formed (*swr1Δ*), if it forms but cannot bind to chromatin (*swc2Δ*) or if it binds to chromatin but lacks histone replacement activity (*swc5Δ* and the ATPase-dead *swr1-K727G*). These results suggest that in *htz1Δ* the nucleosome remodelling activity of SWR1 affects chromatin integrity because of an attempt to replace H2A with Htz1 in the absence of the latter. This would impair transcription and, either directly or indirectly, other cellular processes. Specifically, we show that in *htz1Δ*, the SWR1 complex causes an accumulation of recombinogenic DNA damage by a mechanism dependent on phosphorylation of H2A at Ser129, a modification that occurs in response to DNA damage, suggesting that the SWR1 complex impairs the repair of spontaneous DNA damage in *htz1Δ*. In addition, SWR1 causes DSBs sensitivity in *htz1Δ*; consistently, in the absence of Htz1 the SWR1 complex bound near an endonuclease HO-induced DSB at the mating-type (*MAT*) locus impairs DSB-induced checkpoint activation. Our results support a stepwise mechanism for the replacement of H2A with Htz1 and demonstrate that a tight control of this mechanism is essential to regulate chromatin dynamics but also to prevent the deleterious consequences of an incomplete nucleosome remodelling.

## Introduction

Genome organization and function rely on the precise assembly and dynamics of chromatin. The repeating unit of chromatin, the nucleosome, is formed by 146 bp of DNA wrapped twice around an octamer of histones. Histones H3 and H4 are first assembled into a core (H3/H4)_2_ tetramer, which is the stable entity at physiological ionic strength. An H2A/H2B dimer associates on each side of the tetramer to form the histone octamer that is stabilized by the nucleosomal DNA [1]. ATP-dependent remodelling complexes, histone post-translational modifications and replacement of canonical with variant histones can later modify nucleosomes, thus altering the function of specific chromatin regions [2]–[4].

Variant histones alter the physicochemical properties of nucleosomes and thereby not only the interactions of nucleosomes with other factors but also their stability and DNA accessibility. One such variant, H2A.Z – Htz1 in yeast –, is an evolutionary conserved histone (90% sequence identity across species) with roles in transcription, silencing, genome integrity and cell cycle progression [5], [6]. Htz1 is widely distributed throughout the yeast genome (in more than 65% of the genes) occupying preferentially the nucleosomes flanking the nucleosome-free region located at the transcription start site [7]–[11]. Htz1 is enriched at the promoter of basal/repressed genes where it facilitates transcription activation by histone loss [7]–[13]. Additionally, Htz1 antagonizes silencing by collaborating in the formation of a boundary that prevents the spreading of heterochromatin proteins [14]. These mechanisms, which appear to be conserved in vertebrate cells [15], provide an explanation for the elevated number of down-regulated genes in the absence of Htz1 [14], [16], [17]. The fact that a similar number of genes is up-regulated in *htz1Δ* has also led to proposing a role for Htz1 in repression, even though no evidence has been provided yet.

H2A.Z/Htz1 is also involved in genome stability. It is a structural component of centromeres [18], [19] required for proper chromosome segregation [19], [20]. In addition, the absence of Htz1 affects DNA replication and cell cycle progression and causes lethality or sickness in combination with S-phase checkpoint mutants [21]. These results, together with the sensitivity of *htz1Δ* to drugs causing DNA damage during DNA replication [16], [17], suggest a role for Htz1 in the DNA damage response by replicative stress. Whether or not associated with these phenotypes, Htz1 is transiently recruited to double-strand breaks (DSBs) [22] but its role in DNA repair remains unclear.

H2A.Z/Htz1 is incorporated into chromatin by the Swi2/Snf2-related SWR1 complex [10], [16], [17], [23]. The 14-subunit yeast SWR1 has been extensively characterized *in vitro*. Purified SWR1 complex can specifically replace H2A/H2B with Htz1/H2B in an ATP-dependent manner [17]. Swr1 is the catalytic subunit of the complex and the main scaffold for the assembly of the remaining subunits; Swc5, Swc2, Yaf9 and Arp4 are also required for histone replacement *in vitro*. In addition, Swc2, and less strongly the N-terminal region of Swr1, are the two components that interact directly with Htz1 [24], [25]. Little is known, however, about the mechanisms of SWR1 targeting and Htz1 replacement *in vivo*. It has been shown that Swr1, Yaf9, the bromodomain-containing Bdf1 protein and the module formed by Swc2, Swc6, Arp6 and Swc3 are required for Htz1 incorporation into chromatin [9], [10], [16], [17], [23], but except for Swr1 the specific function of the remaining subunits and therefore the mechanism of replacement are still obscure.

Here we show that in the absence of Htz1 the SWR1 complex causes genetic instability, sensitivity to stress conditions and genome-wide transcriptional misregulation. Our results are consistent with a stepwise mechanism of histone replacement that, in the absence of Htz1, affects chromatin integrity and function.

## Results

### The SWR1 complex causes genetic instability in the absence of Htz1

**Figure 1:**
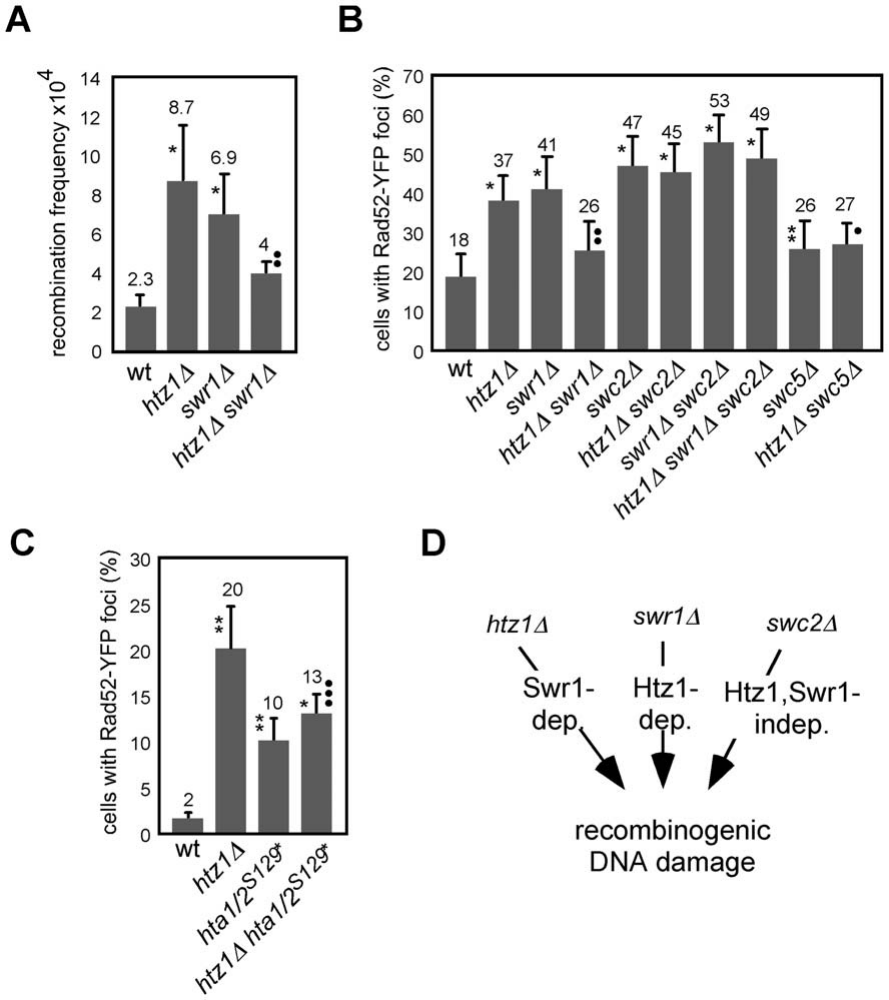
The SWR1 complex causes genetic instability in the absence of Htz1. (**A**) Effect of *htz1Δ*, *swr1Δ* and *htz1Δ swr1Δ* (BY4741) on the frequency of inverted-repeat recombination. (**B**) Effect of *htz1Δ*, *swr1Δ*, *swc2Δ*, *swc5Δ*, *htz1Δ swr1Δ*, *htz1Δ swc2Δ*, *htz1Δ swc5Δ*, *swr1Δ swc2Δ* and *htz1Δ swr1Δ swc2Δ* (BY4741) on the frequency of budded cells with Rad52-YFP foci. (**C**) Effect of *htz1Δ*, hta1/2^S129*^ and htz1Δ hta1/2^S129*^ (W303-1a) on the frequency of budded cells with Rad52-YFP foci. (**D**) Scheme with the pathways of accumulation of recombinogenic DNA damage in *htz1Δ*, *swr1Δ* and *swc2Δ*. The frequency of recombination and budded cells with Rad52-YFP foci is presented as the average and standard deviation as described in Materials and Methods. Asterisks and circles indicate statistically significant differences compared to wild type and *htz1Δ*, respectively, according to a Student's *t*-test, where one asterisk/circle represents a *P*-value <0.0001, two represents <0.005, and three represents <0.025.

To uncover new mechanisms by which chromatin prevents genetic instability, we have analyzed the effects on recombination of mutants affected either in structural components or in remodelling factors of chromatin. This screening revealed a new function for the histone variant Htz1 in preventing the accumulation of recombinogenic DNA damage, as shown by an increase in the frequency of both genetic recombination between inverted repeats and budded cells with foci of the recombination protein Rad52 fused to the yellow fluorescence protein (Rad52-YFP) in *htz1Δ* cells (Figure 1A and B, respectively). As expected by the fact that Swr1 is required for the incorporation of Htz1 into chromatin [17], [23], the absence of Swr1 led to similar phenotypes (Figure 1A and B). Notably, however, *htz1Δ swr1Δ* displayed levels of genetic recombination and Rad52-YFP foci close to the wild type (Figure 1A and B). These results therefore support the existence of two pathways that lead to an accumulation of recombinogenic DNA damage, one associated with *htz1Δ* that depends on Swr1, and another associated with *swr1Δ* that depends on Htz1 (Figure 1D).

The effect of Swr1 in *htz1Δ* is likely to be mediated by the SWR1 complex because SWR1 remains intact in the absence of Htz1 [24]. Given that Swr1 is essential for the integrity of the complex, we decided to study genetic stability in the absence of either Swc2 or Swc5, two SWR1 subunits required for Htz1 transfer *in vitro* but not for the integrity of the complex [24]. Swc2 binds directly to Htz1 and this interaction is responsible for most of the Htz1 bound to the complex. By contrast, Swc5 is the only subunit absent in the complex purified from *swc5Δ* cells [24], [25] and therefore a convenient mutant to explore if the phenotypes mediated by Swr1 in *htz1Δ* require the histone transfer activity of the SWR1 complex.

The absence of Swc2 increased the proportion of budded cells with Rad52-YFP foci (Figure 1B; [26]). However, this increase was also detected in the triple mutant *htz1Δ swr1Δ swc2Δ*, despite the fact that the double mutant *htz1Δ swr1Δ* does not accumulate Rad52-YFP (Figure 1B), supporting the existence of a *swc2Δ*-associated mechanism leading to Rad52-YFP foci that is independent of Swr1 and Htz1 (Figure 1D). By contrast, *swc5Δ* caused just a slight increase in the frequency of cells with Rad52-YFP foci, and more importantly, *swc5Δ* partially suppressed the hyper-recombination phenotype of *htz1Δ* (Figure 1B).

Phosphorylation of H2A is one of the earliest molecular events in response to DNA damage [27], [28] that is required for SWR1 binding to H2A *in vitro* [29] and to damaged DNA *in vivo* [30], [31]. Therefore, we analyzed the effect of a H2A mutant that cannot be phosphorylated (hta1/2^S129*^) on *htz1Δ*-induced genetic instability. As shown in Figure 1C, hta1/2^S129*^ displayed a 5-fold increase in cells with Rad52-YFP foci, consistent with its defect in NHEJ but not in HR [28], but two-fold lower than in *htz1Δ*. Importantly, the effect of hta1/2^S129*^ was epistatic over *htz1Δ*, indicating that the accumulation of Rad52-YFP in *htz1Δ* requires phosphorylation of H2A.

### The SWR1 complex causes DNA damage sensitivity in the absence of Htz1

**Figure 2:**
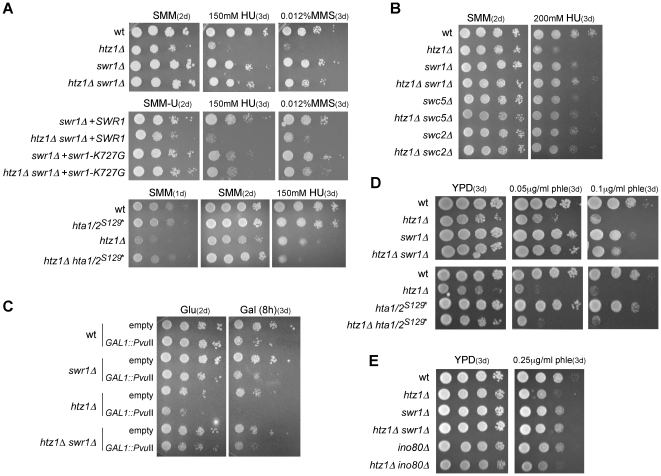
The SWR1 complex causes DNA damage sensitivity in the absence of Htz1. (**A**) DNA damage sensitivity of *htz1Δ*, *swr1Δ*, *htz1Δ swr1Δ*, hta1/2^S129*^, htz1Δ hta1/2^S129*^ and of *swr1Δ* and *htz1Δ swr1Δ* transformed with plasmids pRS416-SWR1-2Flag or p416-swr1-2Flag-k727G (W303-1a) as determined by plating ten-fold serial dilutions from the same number of mid-log phase cells onto SMM or SMM-U plates with or without HU or MMS, respectively. (**B**) DNA damage sensitivity of *htz1Δ*, *swr1Δ*, *swc2Δ*, *swc5Δ*, *htz1Δ swr1Δ*, *htz1Δ swc2Δ* and *htz1Δ swc5Δ* (BY4741) as indicated in (A). (**C**) Sensitivity of *htz1Δ*, *swr1Δ* and *htz1Δ swr1Δ* (W303-1a) to DSBs generated by expression of the endonuclease *Pvu*II from a *GAL1* promoter variant. DSB sensitivity of cells transformed with either pV10 (*GAL1*pr::*Pvu*II) or pRS316 (empty vector) was determined by plating onto SMM-U ten-fold serial dilutions from the same number of mid-log phase cells growth under non-inducing (glucose) or inducing (galactose for 8 hours) conditions. (**D**) DSB sensitivity of *htz1Δ*, *swr1Δ*, *htz1Δ swr1Δ*, hta1/2^S129*^ and htz1Δ hta1/2^S129*^ (W303-1a) as determined by plating ten-fold serial dilutions from the same number of mid-log phase cells onto YPD with or without phleomycin. (**E**) Phleomycin-induced DSBs sensitivity of *htz1Δ*, *swr1Δ*, *htz1Δ swr1Δ*, *ino80Δ* and *htz1Δ ino80Δ* (BY4733) as determined in (D). Cells were incubated at 30°C for 1–3 days as indicated.

The response to DNA damage is not similar in *htz1Δ* and *swr1Δ* cells. While *htz1Δ* is highly sensitive to the replication inhibitor hydroxyurea (HU) and the alkylating agent methyl methanesulfonate (MMS), *swr1Δ* is either resistant or moderately sensitive depending on the genetic background (Figures 2A and S1A; [16], [17]). Since the high density of cells in the drop-test assays can exacerbate growth defects and does not distinguish between lethality and slow growth, we determined the efficiency of plating in media with drug relative to the controls without drug. At the analyzed concentrations *htz1Δ* cells died in HU and grew slowly in MMS (Figure S2), consistent with the fact these two agents cause different types of replicative DNA damage. We hypothesized that DNA damage sensitivity in *htz1Δ* might be mediated by Swr1. In agreement with this possibility we observed that *swr1Δ* suppresses – totally or partially depending on the genetic background – *htz1Δ*-mediated DNA damage sensitivity (Figures 2A, S1A and S2), further demonstrating that Swr1 leads to genetic instability in the absence of Htz1. Also, we observed that both *swc2Δ* and *swc5Δ*, while displaying the same low sensitivity to HU as *swr1Δ*, suppressed *htz1Δ* lethality (Figures 2B and S2A).

These results suggest that DNA damage sensitivity in the absence of Htz1 requires the histone exchange activity of SWR1. To further support this point we analyzed a mutant carrying a K to G substitution in the ATP binding site of Swr1 that completely abolish its histone replacement activity but has no effect on the integrity of the complex [17]. We transformed *swr1Δ* and *htz1Δ swr1Δ* mutants with a plasmid expressing either *SWR1* or *swr1-K727G* and tested their sensitivity to HU and MMS. Expression of the wild-type but not of the Swr1 ATPase-dead protein in *htz1Δ swr1Δ* caused DNA damage sensitivity, indicating that the histone replacement activity of SWR1 is responsible for genetic instability in the absence of Htz1 (Figures 2A and S2A). Finally, and in contrast to the accumulation of Rad52 foci, *htz1Δ* sensitivity to HU was independent of H2A phosphorylation (Figure 2A).

In response to a DSB the SWR1 complex incorporates Htz1 at the proximity of the break [22], [32]. Therefore, we decided to determine whether Swr1 also lead to defective DSB repair in the absence of Htz1, as shown above for HU and MMS. To assess this possibility we first determined the sensitivity of mutant and wild type cells to DSBs generated by the endonuclease *Pvu*II expressed from a *GAL1* promoter variant with reduced basal activity [33]. While the growth of wild type and *swr1Δ* was not affected by the residual expression of *Pvu*II under non-inducing conditions and equally affected upon induction of the *GAL1* promoter for 8 hours, *htz1Δ* was highly sensitive even under residual *PvuII* expression. Remarkably, *swr1Δ* suppressed *htz1Δ* sensitivity to DSBs under both conditions (Figures 2C and S1B). Similar results were obtained with the DSB-inducing drug phleomycin; *htz1Δ* but not *swr1Δ* was highly sensitive to the drug and this sensitivity was partially or totally suppressed – depending on the genetic background – in the double mutant (Figure 2D and S1B). Further analysis showed that phleomycin caused a loss of viability in *htz1Δ* (Figure S2A). These data indicate that the absence of Htz1 at chromatin has a minor impact in DSB repair and that Swr1 causes sensitivity to DSBs in *htz1Δ*. However, and despite the fact that SWR1 has been shown to be recruited to DSBs via P-H2A [31], the phosphoacceptor mutant hta1/2^S129*^ did not suppress *htz1Δ* sensitivity to phleomycin (Figure 2D).

Since SWR1 is closely related to the chromatin remodelling complex INO80 and both SWR1 and INO80 are recruited to DSBs [29]–[31], [34], where they appear to regulate the level of Htz1 [32], we decided to address the possibility that the effect of SWR1 on DSB repair in *htz1Δ* was mediated by INO80. Figure 2E shows that both *swr1Δ* and *ino80Δ* displayed a similar low sensitivity to phleomycin-induced DSBs, consistent with the minor role, if any, played by SWR1 and INO80 in NHEJ and DSB-induced HR [31], [32]. Unlike *swr1Δ*, however, *ino80Δ* did not suppressed *htz1Δ* sensitivity, indicating that INO80 is not required for SWR1-dependent *htz1Δ* sensitivity to DSBs. Altogether, our results, performed in two genetic backgrounds, demonstrate that SWR1 causes sensitivity to a number of different DNA lesions.

### The SWR1 complex impairs DSB-induced checkpoint activation in the absence of Htz1

**Figure 3:**
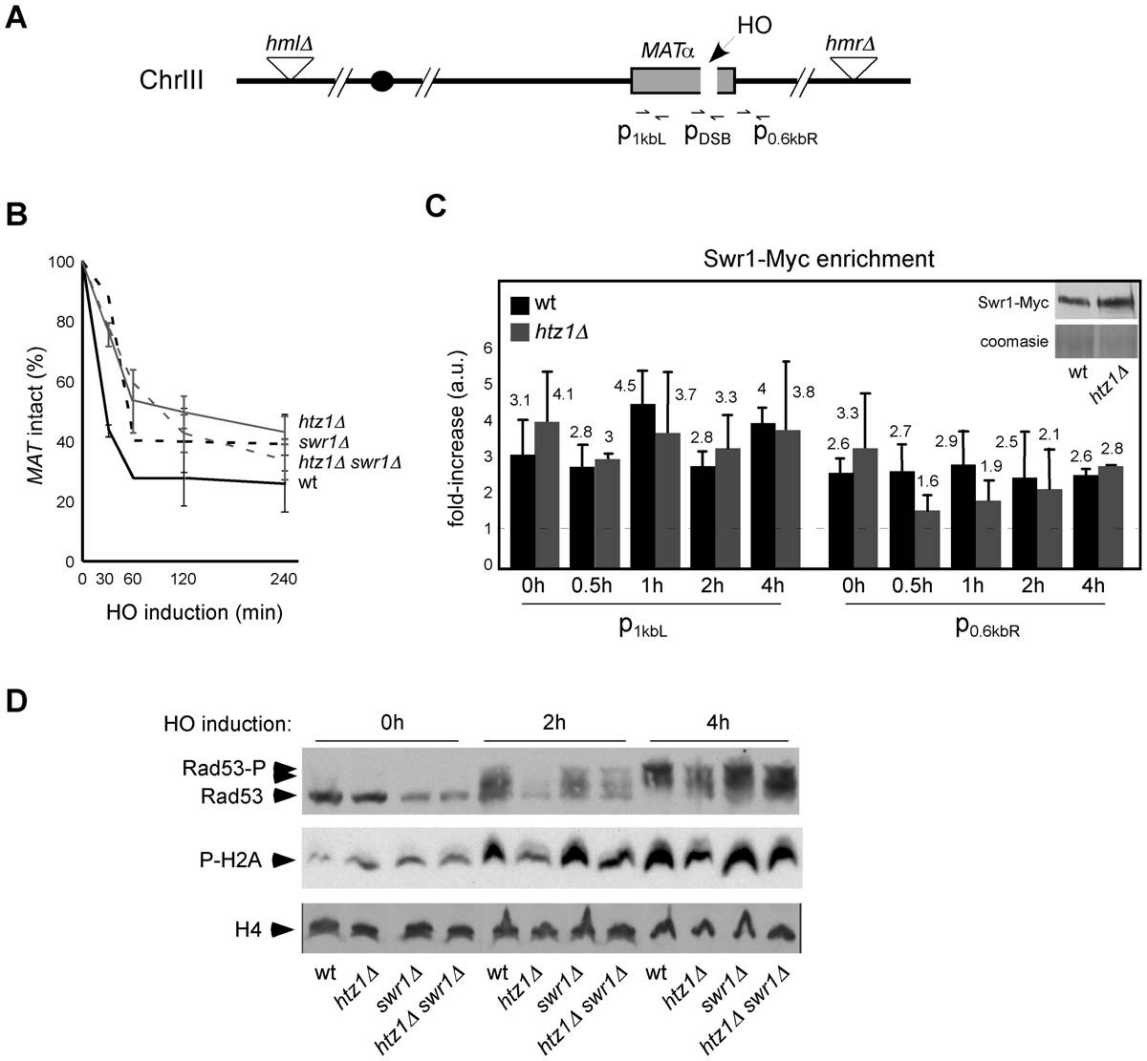
The SWR1 complex impairs DSB processing in the absence of Htz1. (**A**) Schematic representation of the *MAT* locus and deleted *HML* and *HMR* donor sequences at chromosome III. The position of the DNA fragments amplified by qPCR for cleavage efficiency and ChIP analysis is indicated. (**B**) Accumulation of HO-induced DSBs at *MAT* over time. (**C**) Swr1-Myc enrichment at both sides of the cleavage site at *MAT* by ChIP analysis. Both input and ChIP DNA were amplified by real-time PCR with amplicons situated at the regions shown in (A) (see Table S4 for oligos). The enrichment at each zone is graphed relative to the enrichment in the wild-type strain incubated with IgG, taken as 1. Similar results were obtained using as a control the untagged strain incubated with the anti-Myc antibody. (**D**) DSB-induced phosphorylation of Rad53 (Rad53-P) and histone H2A (P-H2A) over HO digestion by western blot. All the experiments were performed in JKM179.

To understand why SWR1 affects DSB repair in *htz1Δ* we first analyzed by ChIP the binding of the SWR1 complex to an induced DSB using a Myc-tagged version of Swr1. We used a yeast strain in which an unrepairable DSB at the mating-type (*MAT*) locus can be synchronously generated by continuous expression of the endonuclease HO from the galactose-inducible *GAL1* promoter, and in which the deletion of the donor sequences *HML* and *HMR* prevents the repair of the DSB by HR (Figure 3A; [35]). The efficiency of HO-induced cleavage at *MAT* was reduced as compared with previous results due to growth conditions (minimal versus rich medium; data not shown) and slightly affected in *htz1Δ*, *swr1Δ* and *htz1Δ swr1Δ* (Figure 3B; [22]). As shown in Figure 3C, and in contrast to a previous result [31], Swr1 was present at *MAT* before formation of the DSB and this binding was not altered over HO digestion. The accumulation of SWR1 at *MAT* before cleavage was not due to incomplete repression of the *GAL1* promoter in raffinose because a similar enrichment was detected in glucose (data not shown). Also, Swr1 binding to *MAT* before and after DSB formation was not affected in *htz1Δ*, indicating that the absence of Htz1 has no effect on SWR1 binding to intact and broken DNA molecules.

It is well established that during DSB repair the 5′-ends generated upon the break are resected leaving 3′-ended single-strand DNA molecules that trigger the activation of the DNA damage checkpoint [36]. Notably, processing of the break has been shown to be different in *htz1Δ* and *swr1Δ* mutants. DNA resection and checkpoint activation – as determined by accumulation of phosphorylated H2A (P-H2A) and Rad53 (P-Rad53) – are affected during HO endonuclease-induced DSB repair in *htz1Δ* [22] but not in *swr1Δ* [31], [32]. We hypothesized that SWR1 might impair DSB processing in the absence of Htz1. To address this possibility we followed the accumulation of P-H2A and P-Rad53 in response to an unrepairable DSB at the *MAT* locus (Figure 3D). As previously shown, the kinetics of Rad53 and histone H2A phosphorylation were not affected in *swr1Δ* and delayed in *htz1Δ* despite equivalent cleavage efficiencies. More importantly, the absence of Swr1 suppressed the defect in checkpoint activation associated with *htz1Δ* (Figure 3D).

### Swr1 causes sensitivity to stress conditions in *htz1Δ*

**Figure 4:**
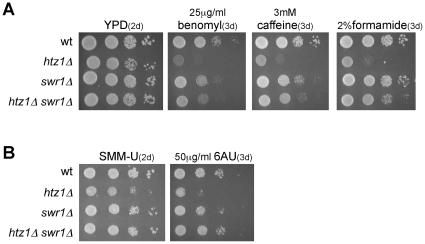
Swr1 causes sensitivity to stress conditions in the absence of Htz1. Stress sensitivity of *htz1Δ*, *swr1Δ* and *htz1Δ swr1Δ* (W303-1a) as determined by plating ten-fold serial dilutions from the same number of mid-log phase cells onto YPD plates with or without benomyl, caffeine or formamide, or SMM without uracil (SMM-U) plates with or without 6-AU. Cells were incubated at 30°C for 2–3 days as indicated.

In addition to DNA damage, *htz1Δ* and *swr1Δ* have been reported to be sensitive to stress conditions that impair different cell processes. In particular, they are sensitive to the microtubule polymerization inhibitor benomyl, a result consistent with their genetic interactions with mutations in components of the kinetochore and the Swr1-mediated deposition of Htz1 at centromeric regions [19]. However, this sensitivity is more pronounced in *htz1Δ* than in *swr1Δ* (Figures 4A; [16], [19]). The absence of Htz1 has also been shown to cause sensitivity to the denaturing agent formamide and to the phosphatidylinositol-3-OH kinase related kinases (PIKKs) inhibitor caffeine, these sensitivities also being high in *htz1Δ* and moderate in *swr1Δ* (Figures 4A [16]). Finally, the absence of both Htz1 and components of SWR1 display synthetic growth defects with the absence of the transcriptional elongation factor Dst1 [23], a result that, together with the sensitivity of *htz1Δ* to the transcriptional elongation inhibitor 6-azauracil (6-AU) [37], points to a role for Htz1 during transcription elongation. We observed that *htz1Δ* sensitivity to 6-AU was less pronounced in *swr1Δ* than in *htz1Δ* (Figure 4B). In all cases, however, the differential sensitivity to drugs of *htz1Δ* as compared to *swr1Δ* was dependent on genetic background (compare Figure 4 with S1C). In addition, these drugs did not lead to a significant loss of viability and only in the case of formamide to a dramatic slow growth, being the growth defects exacerbated by the drop-test assay (Figure S2). More importantly, our study in two different genetic backgrounds showed that the absence of Swr1 suppresses the growth defects of *htz1Δ* under all tested stress conditions, including unperturbed conditions (Figures 4, S1C and S2).

### The SWR1 complex causes transcriptional misregulation in the absence of Htz1

**Figure 5:**
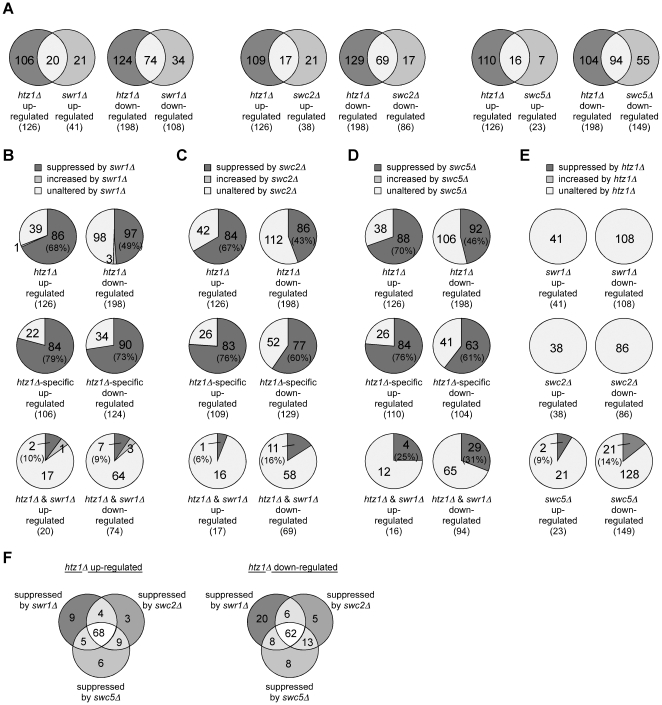
The SWR1 complex causes transcriptional misregulation in the absence of Htz1. (**A**) Venn diagrams showing the number of genes whose mRNA levels changed by more than 2-fold in mutants relative to the wild type and the number of genes that were commonly misregulated by *htz1Δ* and either *swr1Δ*, *swc2Δ* or *swc5Δ*. (**B, C, D**) Fraction of *htz1Δ* misregulated genes (2-fold cutoff) whose mRNA levels were changed by more than 1.5-fold (either suppressed or increased) in the double mutant *htz1Δ swr1Δ* (B), *htz1Δ swc2Δ* (C) or *htz1Δ swc5Δ* (D) relative to *htz1Δ*. (**E**) Fraction of *swr1Δ*, *swc2Δ* or *swc5Δ* misregulated genes (2-fold cutoff) whose mRNA levels changed by more than 1.5-fold in *htz1Δ swr1Δ*, *htz1Δ swc2Δ* or *htz1Δ swc5Δ* relative to *swr1Δ*, *swc2Δ* or *swc5Δ*, respectively. (**F**) Venn diagrams showing the number of *htz1Δ*-misregulated genes (2-fold cutoff) that were commonly suppressed by *swr1Δ*, *swc2Δ* and *swc5Δ*. The genome-wide transcriptional analysis was performed in BY4741.

A number of studies have provided strong genetic and molecular evidence for a function of Htz1 in transcription regulation [7], [9], [10], [14], [17], [23]. Interestingly, comparison of the genome-wide transcription profiles for *htz1Δ* and *swr1Δ* showed a high percentage of genes whose misregulation was specific to each mutation, a result that suggested the existence of non-overlapping functions for Swr1 and Htz1. This effect was particularly relevant in the case of *htz1Δ* with percentages of 86% and 64% of the total amount of *htz1Δ* up- and down-regulated genes, respectively [17]. In light of our previous results we decided to explore the possibility that Swr1 caused transcriptional misregulation in *htz1Δ*. With this aim the transcription profiles of single and double mutants and wild-type cells were determined by whole-genome microarray analysis (Figure 5 and Tables S1 and S2). A 2-fold expression change cutoff relative to wild type yielded 126 and 41 up-regulated genes and 198 and 108 down-regulated genes by *htz1Δ* and *swr1Δ*, respectively (Figure 5A). Overall these numbers are 10–20-fold higher than those obtained by Mizugushi and co-workers with the same strains but in a rich medium [17], indicating that the unexpected low number of genes regulated by Swr1 and Htz1, considering that Htz1 is present in most yeast promoters, was mostly due to growth conditions. Apart from this effect and consistent with the spreading of heterochromatin proteins in the absence of Htz1 incorporation into chromatin [14], 25% of the genes down-regulated in *htz1Δ* accumulated near telomeres in small clusters (*H*tz1-*a*ctivated *d*omains; HZADs) and 60% of these genes were also down-regulated by *swr1Δ* (Table S2).

A comparative analysis of *htz1Δ* and *swr1Δ* transcriptional profiles shows that the number of misregulated genes was much higher in *htz1Δ* than in *swr1Δ*. Also, most of the genes misregulated by *htz1Δ* were not misregulated by *swr1Δ* (84% and 63% for up- and down-regulated), as opposed to the number of genes misregulated by *swr1Δ* that were not misregulated by *htz1Δ* (51% and 31% for up- and down-regulated) (Figure 5A). More importantly, most of the *htz1Δ*-misregulated genes were suppressed by more than 1.5-fold by *swr1Δ* (68% and 49% of the up- and down-regulated genes) (Figure 5B) without a preferential association with genes located at HZADs or randomly distributed (Table S2). It is noted that this suppression by *swr1Δ* mainly affected the genes that were specifically misregulated by *htz1Δ* (79% and 73% for up- and down-regulated, respectively) and not the genes misregulated by both *htz1Δ* and *swr1Δ* (10% and 9% for up- and down-regulated, respectively) (Figure 5B). By contrast, the changes in the level of mRNA caused by the absence of Swr1 were not significantly affected by *htz1Δ* (Figure 5E). These results indicate that Swr1 affects the expression of a large number of genes in the absence of Htz1, and suggest that the major role for Htz1 in transcription is mediated by Swr1. Similar results were obtained with 1.5-fold-expression and 1.2-fold-suppression cutoffs (Tables S1 and S2).

Next, we decided to determine whether transcriptional misregulation in the absence of Htz1 was also dependent on Swc2 and Swc5. As shown in Figure 5A, the number of genes up- and down-regulated either by *htz1Δ* alone or *htz1Δ* and *swc2Δ* (or *swc5Δ*) together were similar to those obtained with either *htz1Δ* alone or *swr1Δ* and *htz1Δ* together, respectively. Also, these groups of genes displayed a significant overlapping (Figures S3A and S3B). Importantly, *swc2Δ* and *swc5Δ* suppressed a similar (Figure 5C and 5D) and common (Figure 5F) number of *htz1Δ*-misregulated genes as did *swr1Δ*, indicating that the SWR1 complex impairs transcription in the absence of Htz1 and that the major role of Htz1 in transcription occurs via SWR1-mediated histone replacement. It is also noted that misregulation by *swc2Δ* and *swc5Δ* was not affected by the absence of Htz1, except for a reduced number of genes (23 out of 172) whose *swc5Δ*-mediated change in mRNA levels was suppressed by *htz1Δ* (Figure 5E and Table S1), and that may reflect a residual activity of the SWR1swc5Δ complex. Interestingly, the genes misregulated specifically by the absence of Swr1, Swc2 or Swc5 do not show a significant overlapping (Figure S3C), suggesting that the SWR1 complex does not have a physiological role in transcription regulation independent of Htz1.

### Swc2, but not Swc5 and Htz1, is required for Swr1 binding to chromatin, while Swc5 is required for histone replacement

**Figure 6:**
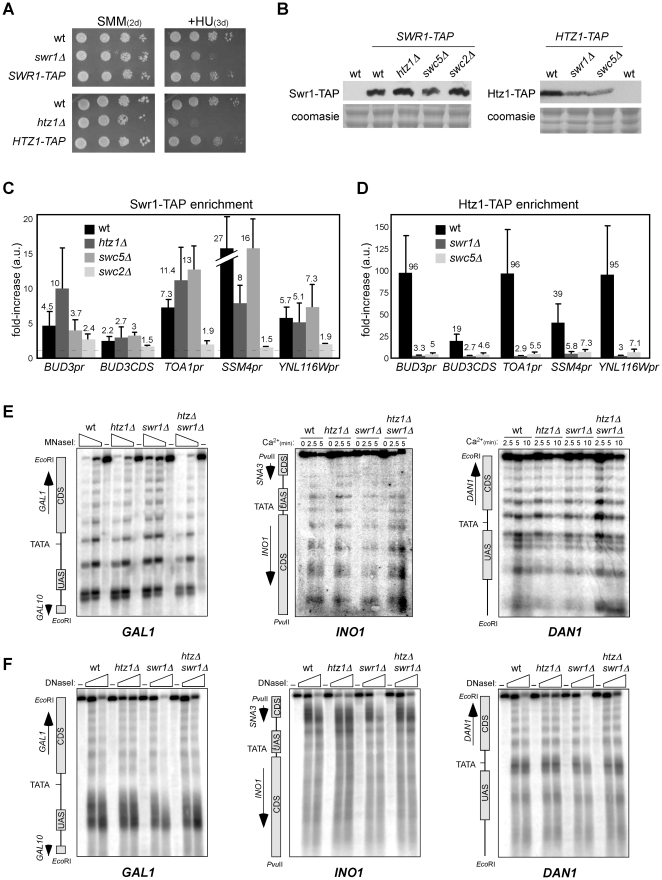
Swc2 and Swc5 are required for Swr1 binding to chromatin and histone replacement, respectively. (**A**) Swr1-TAP and Htz1-TAP functionality as determined by plating ten-fold serial dilutions from the same number of mid-log phase cells onto SMM plates with or without 200 mM HU. Cells were incubated at 30°C for 2–3 days as indicated. (**B**) Swr1-TAP and Htz1-TAP protein levels in mutants and wild type as determined by western blot. (**C**) Swr1-TAP and (**D**) Htz1-TAP enrichment at the promoters of *BUD3*, *TOA1*, *SSM4* and *YNL116w* and an internal region of the coding sequence of *BUD3* by ChIP analysis. Both input and ChIP DNA from untagged and tagged cells were amplified by real-time PCR with amplicons situated at the indicated regions (see Table S4 for oligos). The enrichment in the tagged strain at each zone is graphed relative to the enrichment in the untagged strain, taken as 1. ChIP experiments were performed in BY4741. (**E**) Nucleosome positioning at *GAL1* in W303-1a and *INO1* and *DAN1* in BY4741 as determined by MNaseI digestion and indirect-end labelling. Spheroplasts from W303-1a cells were treated with different amounts of MNaseI, while BY4741 cells expressing the MNaseI were incubated with Ca^2+^ to activate the nuclease for the indicated times. (**F**) DNA accessibility at the genes indicated in (E) by DNaseI digestion and indirect-end labelling. A scheme with the position of the upstream activation (UAS) and coding sequences (CDS) is shown on the left of each panel.

Our previous results indicate that genetic instability, sensitivity to stress and transcriptional misregulation in *htz1Δ* are, to a greater or lesser extent, the consequence of the activity of the SWR1 complex. To get better insight into this mechanism we decided to determine what steps of the histone replacement reaction were prevented in our mutants. First, we analyzed SWR1 targeting to chromatin by ChIP analysis of strains harbouring a TAP-tagged version of Swr1. This construct is functional as indicated by the fact that *SWR1-TAP* displayed the same resistance to HU as the wild type (Figure 6A). We chose the promoter and an internal region of the *BUD3* gene known to be enriched or not in Htz1, respectively [9], and three Swr1 enriched promoters (*TOA1*, *SSM4* and *YNL116w*) [10]. As can be seen in Figure 6C, Swr1-TAP bound to chromatin in wild-type cells and, as shown above for the *MAT* locus, this binding did not require Htz1 (similar results were obtained with Myc-Swr1 binding to the *TOA1* promoter in samples of Figure 3C; Figure S4A). Similarly, Swr1 binding to chromatin was not prevented by the absence of Swc5. However, Swr1 binding to promoters was impaired in *swc2Δ* despite this strain displaying wild-type levels of Swr1-TAP as determined by western analysis (Figure 6B).

Swc5 has been shown to be required for histone transfer *in vitro* [24]. We decided to determine whether Swc5 is also required for Htz1 transfer *in vivo* by ChIP analysis in strains harbouring a functional TAP-tagged version of Htz1, as determined by HU and 6-AU sensitivity (Figure 6A; [23]). Figure 6D shows that Htz1 was not incorporated into chromatin in the absence of Swr1, consistent with previous results [17], [23], while the absence of Swc5 nearly eliminated the amount of Htz1 bound to chromatin. Western blot analysis showed that the amount of Htz1-TAP in both mutants was 3–4-fold lower than in the wild type (Figure 6B) despite the levels of mRNA not being affected (data not shown), suggesting that Htz1 incorporation into chromatin prevents its degradation. Our molecular analysis demonstrates that Swc2 is required to target the SWR1 complex to chromatin, while Swc5 participates in the reaction of histone replacement. Altogether, our results let us conclude that genetic instability and transcriptional misregulation in the absence of Htz1 require the binding to chromatin (prevented in *swc2Δ*) and the histone replacement (prevented in *swc5Δ* and *swr1-K727G*) activities of SWR1.

The requirement for the histone replacement activity of SWR1 prompted us to look for an alteration in chromatin structure associated with *htz1Δ* and to determine its dependence on Swr1. The analysis of nucleosome positioning and DNA accessibility by MNaseI and DNaseI digestion, respectively, of the *INO1*, *DAN1* and *GAL1* promoters did not reveal any difference in chromatin structure between the wild type, *htz1Δ*, *swr1Δ* and *htz1Δ swr1Δ* (Figures 6E and 6F). While a previous work reported a 20 bp shift in the positioning of nucleosome +2 of the *GAL1* promoter in *htz1Δ* with a similar approach, this result was obtained with a wild type strain in which Htz1 was tagged with Myc [7]. We also tried to determine by ChIP analysis if the absence of Htz1 altered the stoichiometry of the nucleosomal histones at three promoters enriched in Htz1, and we did not detect any modification in the H2B/H3 ratio (Figure S4B). Overall our results are in line with both an independent study of four different chromatin regions [8] and a recent genome-wide analysis on nucleosome positioning [38] that have not detected any effect of *htz1Δ* on chromatin structure.

## Discussion

The genome-wide distribution of Htz1 and its role in a number of processes from transcription and silencing to DNA repair and chromosome segregation make of this histone a key regulator of the genome dynamics. Here we show that genetic instability, sensitivity to drugs impairing different cellular processes and genome-wide transcription misregulation in *htz1Δ* can be partially or totally suppressed if the SWR1 complex is not assembled (*swr1Δ*), if it is assembled but cannot bind to chromatin (*swc2Δ* and hta1/2^S129*^ in case of recombinogenic DNA damage) or if it can bind to chromatin but lacks histone replacement activity (*swc5Δ* and *swr1-K727G*), indicating that in the absence of Htz1 the nucleosome remodelling activity of SWR1 affects transcription and genetic stability.

The mechanism of histone replacement has been suggested to occur in a stepwise manner [17] even though these steps remain unknown. We show that Swc2 is required for Swr1 binding to chromatin, and that this binding is not mediated by its interaction with Htz1 because it is not prevented by *htz1Δ*. These results, together with the fact that the mouse homolog of Swc2 (YL-1) binds to DNA *in vitro* [39] point to Swc2 being the subunit that targets SWR1 to chromatin. Upon binding, the SWR1 complex may promote histone replacement in a two-step manner, with the destabilization of H2A/H2B followed by deposition of Htz1/H2B via interactions with Swc2 and Swr1. It has been proposed that SWR1, as shown for SWI/SNF, may generate a dynamic DNA loop on the nucleosomal surface that promotes the intrinsic tendency of the histone octamer to dissociate the H2A/H2B dimer [17]. *In vitro* studies have shown that Swc5 is required for histone replacement [24]; our *in vivo* results support this conclusion and suggest that it is required for the destabilization of the H2A/H2B dimer. In this regard, the presence in Swc5 of a 60-residue N-terminal domain highly enriched in acidic amino acids (43%) characteristic of histone chaperones might provide the binding module to capture the H2A/H2B dimer as shown for Swi3 in SWI/SNF [40]. In a second step, Swr1 and Swc2 may deposit Htz1/H2B thus restructuring the nucleosome.

In this context, the fact that in *htz1Δ* the SWR1 complex causes transcription misregulation and genetic instability by a mechanism that requires both the binding of SWR1 to chromatin and its histone replacement activity led us to propose that in the absence of Htz1 the nucleosome would remain transiently “destabilized” by SWR1 because of an attempt to replace H2A without Htz1, leading to a loss of chromatin integrity and function. However, we note the absence of changes in chromatin structure associated with *htz1Δ* (Figures 6 and S4B; [8], [38]), a result that is consistent with subtle and transient alterations in nucleosome structure and/or a reduced population of affected molecules, but also with SWR1 affecting transcription and genetic stability without altering chromatin structure. For instance, SWR1 might be “trapped” at chromatin becoming a steric hindrance for DNA metabolic processes. However, the fact that the cellular defects associated with *htz1Δ* require the replacement activity of SWR1 (abolished in *swc5Δ* and *swr1-K727G*) and that the enrichment in Swr1 at promoters and broken DNA ends is not significantly higher in *htz1Δ* than in wild type and *swc5Δ* questions this alternative model. Additional analysis will thereby be required to reconcile genetic and molecular data.

Notably, the transcriptional effect of the SWR1 complex is more evident in genes that are specifically misregulated by *htz1Δ*. This result may be explained if we consider gene regulation by histone replacement as a dynamic process whose turnover rate can be low but not absent in those genes that are regulated by SWR1/Htz1 but do not require Htz1 under our experimental growth conditions. The transcriptional defect by *htz1Δ* in those genes would be caused by SWR1; this effect would be masked by the transcriptional defects associated with the absence of Htz1 in the chromatin of those genes that do require regulation by SWR1/Htz1 under our experimental growth conditions. Importantly, our results suggest that the low overlapping of misregulated genes in *swr1Δ* and *htz1Δ* (Figure 5; [17]) is not due to independent functions of these two genes and strengthen the idea that the main role of Htz1 is associated with SWR1. Similarly, our genome-wide transcriptional analysis suggests that SWR1 does not have a prominent role in transcription regulation independent of Htz1. Finally, our results suggest that the sensitivity of *htz1Δ* to drugs impairing different cellular processes is due to, at least in part, the effect of SWR1 on transcription.

We have also shown that in *htz1Δ* the histone replacement activity of SWR1 leads to an accumulation of recombinogenic DNA damage. Notably, this accumulation can be suppressed by a mutation at the H2A phosphoacceptor S129. This is particularly important because H2A phosphorylation is a DNA damage-specific chromatin mark [28], suggesting that the high frequency of Rad52 foci in *htz1Δ* results from a direct effect of SWR1 at DNA lesions. This is also supported by the absence of genes involved in DNA damage repair among those misregulated by *htz1Δ* (Table S2). In addition, the fact that H2A phosphorylation occurs in response to the damage [27], [28] indicates that this accumulation of Rad52 foci is associated with defective DNA repair rather than with the generation of new DNA lesions. In this context it is noteworthy that hta1/2^S129*^ also suppresses the slow growth of *htz1Δ* (Figure 2A), which results from a delayed S phase [21], because points to defects in spontaneous DNA damage repair during DNA replication as a major problem in the absence of Htz1 and account for the synthetic interactions of *htz1Δ* with S-phase but not with DNA-damage checkpoint mutants [21]. These results also suggest that spontaneous DNA lesions leading to HR foci in *htz1Δ* are not DSBs because DSB sensitivity in *htz1Δ* is independent of H2A phosphorylation. Consistent with this, *htz1Δ* cells accumulate P-H2A (data not shown; [32]) despite DSB-induced H2A phosphorylation is retarded (Figure 3), and neither *htz1Δ* nor *swr1Δ* accumulate DSBs as determined by pulse-field genome electrophoresis (Figure S5).

SWR1 and Htz1 have been shown to bind near a DSB [22], [31], [32]. We show that Swr1 is present at *MAT* before formation of a DSB, in contrast to an earlier report [31] but consistent with the Swr1-dependent presence of Htz1 at *MAT* before cleavage detected by two other groups [22], [32]. Normalization to an internal DNA fragment may be responsible for the result obtained by van Attikum and Gasser, since this locus might also be enriched in Swr1. These authors also showed that SWR1 is recruited to sites of DSB after 2–4 hours of HO expression [31]. We found no significant increase in Swr1 binding in response to a DSB, which might be due to differences in growth conditions (minimal versus rich medium). It worth noting, however, that no significant [31], [32] or just a subtle accumulation of Htz1 after 30 minutes of HO expression have been detected [22]. This suggests that Htz1 binding to chromatin in response to DSBs is, at best, slight and transient. Consistently, the function of SWR1/Htz1 at DSBs is unclear, because *htz1Δ*, but not *swr1Δ* is defective in DSB processing, and *swr1Δ* is proficient in HR and only slightly affected in non-homologous end joining (NHEJ) [22], [31]. In agreement with this, we show that *swr1Δ* is hardly sensitive to DSBs. By contrast, SWR1 causes sensitivity to DSBs in *htz1Δ*. Further molecular analysis shows that SWR1 causes a delay in DSB-induced checkpoint activation in *htz1Δ*, likely as a consequence of defects in DNA resection as suggested by the fact that this process is affected in *htz1Δ* but not in *swr1Δ* [22], [31]. As previously mentioned, the absence of DSB repair genes misregulated by *htz1Δ* makes unlikely an indirect effect by transcriptional defects. Another possibility to explain these results would be a direct effect of SWR1 impairing DSB repair in *htz1Δ*; consistent with this idea Htz1 is not required for SWR1 binding to chromatin. Notably, phleomycin-induced DSB sensitivity in *htz1Δ* is not suppressed by hta1/2^S129*^, despite P-H2A has been shown to be required for SWR1 binding in response to a DSB [31], suggesting that the pool of SWR1 at chromatin before the breaks are made is responsible for defective DSB repair. In agreement with this, SWR1 is present at *MAT* before HO cleavage. This situation mimics the role for the INO80 complex at *MAT*, where the pre-existing, but not the P-H2A-dependent pool of INO80 recruited in response to DSBs, is responsible for nucleosome removal from broken ends [41].

In addition to the SWR1-dependent genetic instability in *htz1Δ*, the analysis of mutations in SWR1 subunits have revealed two other mechanisms leading to an accumulation of recombinogenic DNA damage that require further analysis to be understood. The first one occurs in the absence of Swr1 and is mediated by Htz1, while the second occurs in the absence of Swc2 and is independent of Swr1 and Htz1. Whether or not these phenotypes are a consequence of *swr1Δ* (or *swc2Δ*) specific transcriptional defects or are related to unknown mechanisms of genetic instability is well worth addressing.

In summary our results in yeast provide new insights into the mechanism of histone replacement and highlight the importance of a tight control of this process not only to assemble a proper chromatin structure but also to prevent the deleterious consequences of an incomplete nucleosome remodelling. The ample range of cellular defects mediated by the nucleosome remodelling activity of SWR1 in *htz1Δ* prompts us to predict that reductions in the pool of available H2A.Z/Htz1 may have an impact in cell fitness, in particular in the context of the demanding structural complexity of metazoan chromatin. In this regard it is tempting to speculate about the possibility that some of the phenotypes associated with the absence of H2A.Z in metazoan cells, in particular lethality [42], [43], could be influenced by the corresponding SWR1-like complexes.

## Materials and Methods

### Yeast strains, growth conditions and plasmids

Yeast strains used in this study are listed in Table S3. Tagged strains and deletion mutants were constructed by a PCR-based strategy [44]. Yeast cells were grown in supplemented minimal medium (SMM), except for the analysis of benomyl, caffeine, formamide and phleomycin sensitivity, which was performed in YPD rich medium [45]. For the analysis of DSB-induced Swr1-Myc binding to chromatin and checkpoint activation JKM179 derived strains were grown in SMM with 2% raffinose instead of glucose and HO expression was induced by the addition of 2% galactose. pRS316 [46], pRS416-SWR1-2Flag, p416-swr1-2Flag-K727G [17], pRS316-SU [47], pWJ1344 (by R. Rothstein, Columbia University), pV10 [33] and pADS14-nlsMN (by U. K. Laemmli, Geneva University) are centromeric plasmids containing *URA3*, *SWR1*, *swr1-K727G*, the SU inverted repeat recombination system, and the *RAD52-YFP*, GAL1pr*::*Pvu*II and *ADH1pr*::nlsMN constructs, respectively.

### Genetic recombination and DNA damage and stress sensitivity/viability assays

The frequency of Leu^+^ recombinants generated by spontaneous recombination between inverted repeat sequences was determined in cells transformed with plasmid pRS316-SU by fluctuation tests as the median value of six independent colonies [48]. The average and standard deviation of 8 fluctuation tests performed with 4 independent transformants of each strain are shown. DNA damage and stress sensitivity was determined by plating ten-fold serial dilutions from the same number of mid-log phase cells onto medium containing different drugs at the indicated concentrations. Cells were previously transformed either with pV10 or pRS316 for *Pvu*II-mediated DSBs sensitivity and with pRS316 for 6-AU sensitivity. Cell viability in response to DNA damage and stress conditions was determined as the frequency of cells from a colony able to grow in plates containing the different drugs relative to SMM or YPD. The average and standard deviation of 4 independent colonies are shown.

### Analysis of Rad52-YFP foci

The proportion of budded cells with Rad52-YFP foci was performed as described previously [49]. Cells transformed with pWJ1344 were grown to mid-log-phase at 30°C and visualized with a Leica CTR6000 fluorescence microscope. The total numbers of analyzed cells were 600 for *swr1Δ*, *htz1Δ swr1Δ* and *htz1Δ swr1Δ swc2Δ*, 1000 for *swc2Δ*, *swc5Δ*, *htz1Δ swc2Δ* and *swr1Δ swc2Δ*, 1500 for *htz1Δ* and 2500 for *htz1Δ swc5Δ* and the wild type in Figure 1B, and 600 for *htz1Δ*, hta1/2^S129*^, htz1Δ hta1/2^S129*^ and the wild type in Figure 1C. The average and standard deviation of 6–25 independent measures are shown.

### Pulse-field genome electrophoresis (PFGE)

Total DNA from exponentially growing cultures was extracted in low-melting agarose plugs as previously shown [50] and resolved by PFGE (Biorad; 120° field angle; 6 V/cm; 14°C; initial block: switch time of 70 s for 12 h; final block: switch time of 120 s for 16 h).

### Western blot analysis

Yeast protein extracts were prepared using the TCA protocol as described previously [51] and run on a 5%, 7%, 10%, 8% and 15% sodium dodecyl sulfate-polyacrilamyde gel for TAP-Swr1, Myc-Swr1, TAP-Htz1, Rad53 and histones, respectively. TAP constructs were detected by western blot with the rabbit peroxidase anti-peroxidase soluble complex antibody (Sigma). Rad53 was detected with the rabbit polyclonal antibody JDI47 as previously shown [52], Swr1-Myc with the mouse monoclonal antibody MMS-150R against Myc (Covance), and H4 and phosphorylated histone H2A with the rabbit polyclonal antibodies ab10158 and ab15083 (Abcam), respectively.

### HO-induced DSB efficiency

The efficiency of DNA cleavage by HO endonuclease was measured by qPCR on input DNA with oligos spanning the break (p_DSB_) and an uncut control DNA sequence (p_1kbL_) as the ratio p_DSB_/p_1kbL_ in galactose-induced cells relative to that in uninduced cells [31].

### Chromatin immunoprecipitation (ChIP)

ChIP assays were performed as described [53] with the anti-Myc mouse monoclonal antibody ab56 (Abcam) for Swr1-Myc, the rabbit polyclonal antibodies ab13923, ab1790 and ab1791 (Abcam) for H2A, H2B and H3, respectively, and immunoglobulin-sepharose for tandem affinity purification (TAP)-tagged proteins. Oligonucleotide sequences for the real-time PCR amplifications performed on purified DNA before (input; I) or after (immunoprecipitated; IP) immunoprecipitation are shown in Table S4. Protein enrichment at each specific region was calculated as the ratio between the IP and the I in the tagged strain relative to the same ratio either in the untagged strain (for Swr1-TAP and Htz1-TAP) or in the tagged strain incubated with rabbit IgG I8140 (Sigma) (for Swr1-Myc, H2A, H2B and H3). The average and standard deviation of 2–4 independent experiments are shown.

### Chromatin analysis by MNase and DNase digestion

Nucleosome positioning and DNA accessibility were determined by micrococcal nuclease (MNaseI) and DNaseI digestion, respectively, followed by indirect-end labelling. Nucleosome positioning at *GAL1* in W303-1a and DNA accessibility at *GAL1*, *INO1* and *DAN1* were performed by treating spheroplasts with different amounts of MNaseI and DNaseI, respectively, as previously reported [51]. Nucleosome positioning at *GAL1*, *INO1* and *DAN1* in BY4741 was performed with cells previously transformed with plasmid pADS14-nlsMN by *in vivo* ChEC (Chromatin endogenous cleavage) as indicated [54]. MNaseI (or DNaseI)-treated DNA was extracted and restricted with either *Eco*RI (*GAL1* and *DAN1*) or *Pvu*II (*INO1*), resolved in a 1.2% agarose gel, blotted onto a membrane and probed with 200-bp PCR fragments immediately downstream of *Eco*RI (*GAL1* and *DAN1*) or *Pvu*II (*INO1*).

### Microarray hybridization analysis

Gene expression profiles were determined by using the “3′-Expression Microarray” technology by Affymetrix platform at the Genomics Unit of CABIMER (Seville, Spain). Total RNA from yeast cells grown on SMM at 30°C to mid-log phase was isolated using the RNeasy® Midi kit (Qiagen) and its quality confirmed with the Bioanalyzer® (Agilent technology). Synthesis, labelling and hybridization of cRNA to GeneChip® Yeast Genome 2.0 Arrays covering 5841 genes of *S. cerevisiae* was performed with RNA from 3 independent cultures of each strain following Affymetrix recommended protocols (http://www.affymetrix.com/analysis/index.affx). Probe signal intensities were captured and processed with GeneChip Operating Software 1.4.0.036 (Affymetrix), and the resulting CEL files were reprocessed using the Robust Multichip Average (RMA) normalization [55]. Fold-change (log2) values (M) and their FDR-adjusted p-values were calculated with LIMMA (Linear Models for Microarray Analysis) [56] using the *affylmGUI* interface [57]. Limma uses an empirical Bayes method to moderate the standard errors of the estimated log-fold changes. All the statistical analysis was performed using *R* language and the packages freely available from the “Bioconductor Project” (http://www.bioconductor.org). Fold-change cutoffs were analyzed at 95% confidence levels (FDR-adjusted p-values<0.05). All data is MIAME compliant and the raw data have been deposited at the Miame compliant Gene Expression Omnibus (GEO) database at the National Center for Biotechnology Information (http://www.ncbi.nlm.nih.gov/geo/) and are accessible through accession number GSE21571.

## Supporting Information

Figure S1 Swr1 causes DNA damage and stress sensitivity in the absence of Htz1. DNA damage and stress sensitivity as determined in Figures 1 and 2 but in BY4741 strains.
(1.14 MB TIF)

Figure S2 Cell viability (A) and growth (B) in response to DNA damage and stress conditions in W303-1a and BY4741 strains.
(2.04 MB TIF)

Figure S3 Venn diagrams showing the number of genes that were commonly misregulated (2-fold cutoff) by (A) (htz1Δ but no swr1Δ), (htz1Δ but no swc2Δ) and (htz1Δ but no swc5Δ) (B) (htz1Δ and swr1Δ), (htz1Δ and swc2Δ) and (htz1Δ and swc5Δ), (C) (swr1Δ but no htz1Δ), (swc2Δ but no htz1Δ) and (swc5Δ but no htz1Δ) and (D) (htz1Δ swr1Δ), (htz1Δ) and (swr1Δ); (htz1Δ swc5Δ), (htz1Δ) and (swc5Δ); (htz1Δ swc2Δ), (htz1Δ) and (swc2Δ).
(0.19 MB TIF)

Figure S4 (A) Swr1-Myc enrichment at the TOA1 promoter as determined by ChIP analysis of samples in Figure 3C. Both I and IP DNA from cell extracts incubated either with anti-Myc antibody or IgG were amplified by real-time PCR (see Table S4 for oligos). The enrichment is graphed relative to the enrichment in the wild-type strain incubated with IgG, taken as 1. Similar results were obtained using as a control an untagged strain incubated with anti-Myc (data not shown). (B) Histone enrichment at the promoters of BUD3, ARG3 and FIG1 by ChIP analysis. Both I and IP DNA from cell extracts incubated either with anti-H3, anti-H2B, anti-H2A antibodies or IgG were amplified by real-time PCR with amplicons situated at the indicated regions (see Table S4 for oligos). The enrichment is graphed relative to the enrichment in the wild-type strain incubated with IgG, taken as 1. ChIP experiments were performed in BY4741 background.
(0.15 MB TIF)

Figure S5 Analysis of spontaneous DNA breaks as determined by PFGE of yeast chromosomes in htz1Δ, swr1Δ, htz1Δ swr1Δ and wild type.
(0.57 MB TIF)

Table S1 Transcription profiles of htz1Δ relative to swr1Δ, swc2Δ and swc5Δ.
(0.27 MB DOC)

Table S2 Differentially expressed genes in htz1, swr1, swc2, swc5 and double mutants relative to wild type.
(1.06 MB XLS)

Table S3 Strains.
(0.09 MB DOC)

Table S4 Oligos.
(0.05 MB DOC)
