# Supplementary figures and images for: The SWR1 Histone Replacement Complex Causes Genetic Instability and Genome-Wide Transcription Misregulation in the Absence of H2A.Z
Source: PLoS One. 2010 Aug 12;5(8):e12143. doi: 10.1371/journal.pone.0012143 (PMC2920830; doi:10.1371/journal.pone.0012143)

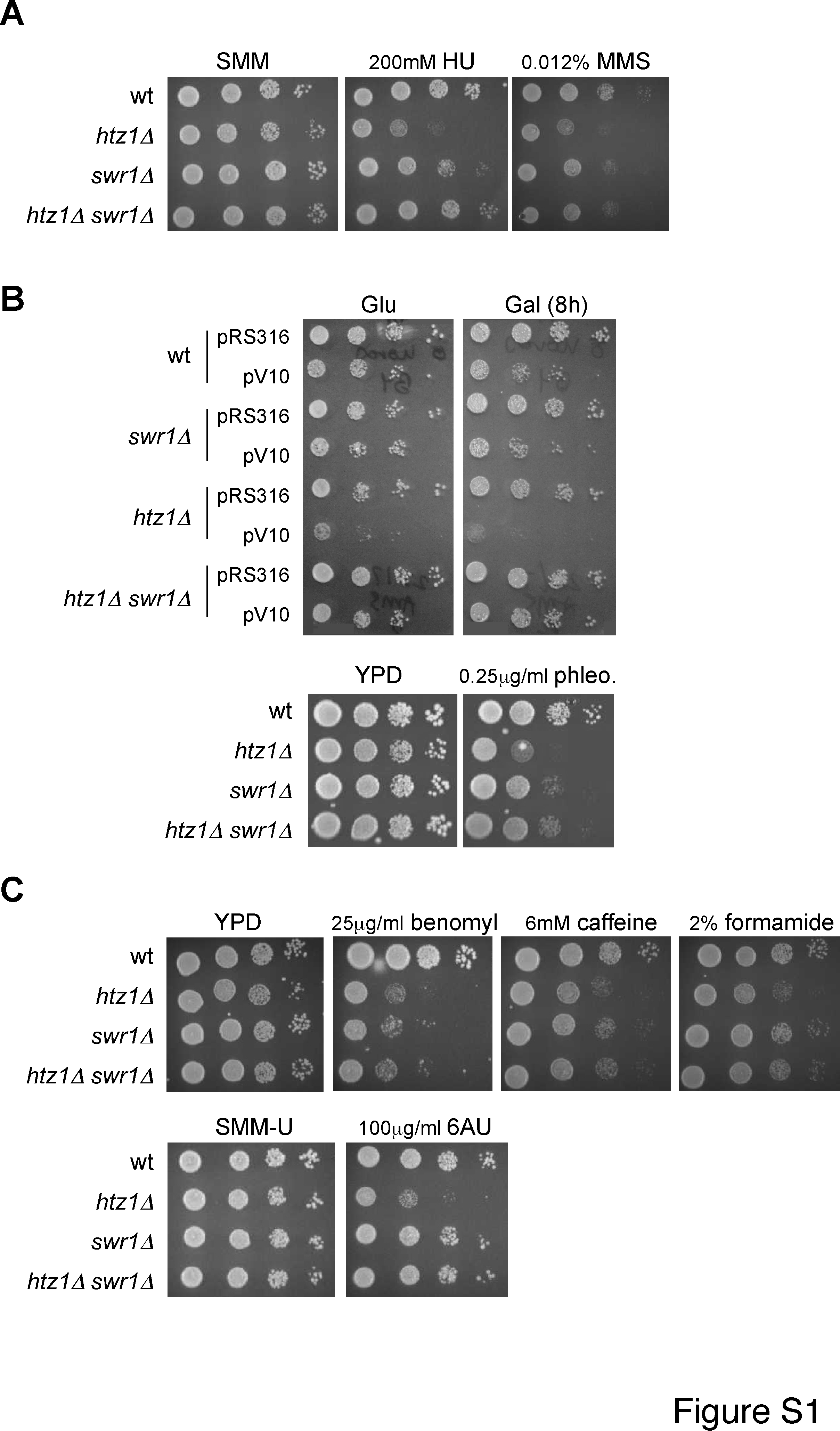

Supplement: Figure S1 — Swr1 causes DNA damage and stress sensitivity in the absence of Htz1. DNA damage and stress sensitivity as determined in Figures 1 and 2 but in BY4741 strains. (1.14 MB TIF) [file pone.0012143.s001.tif]

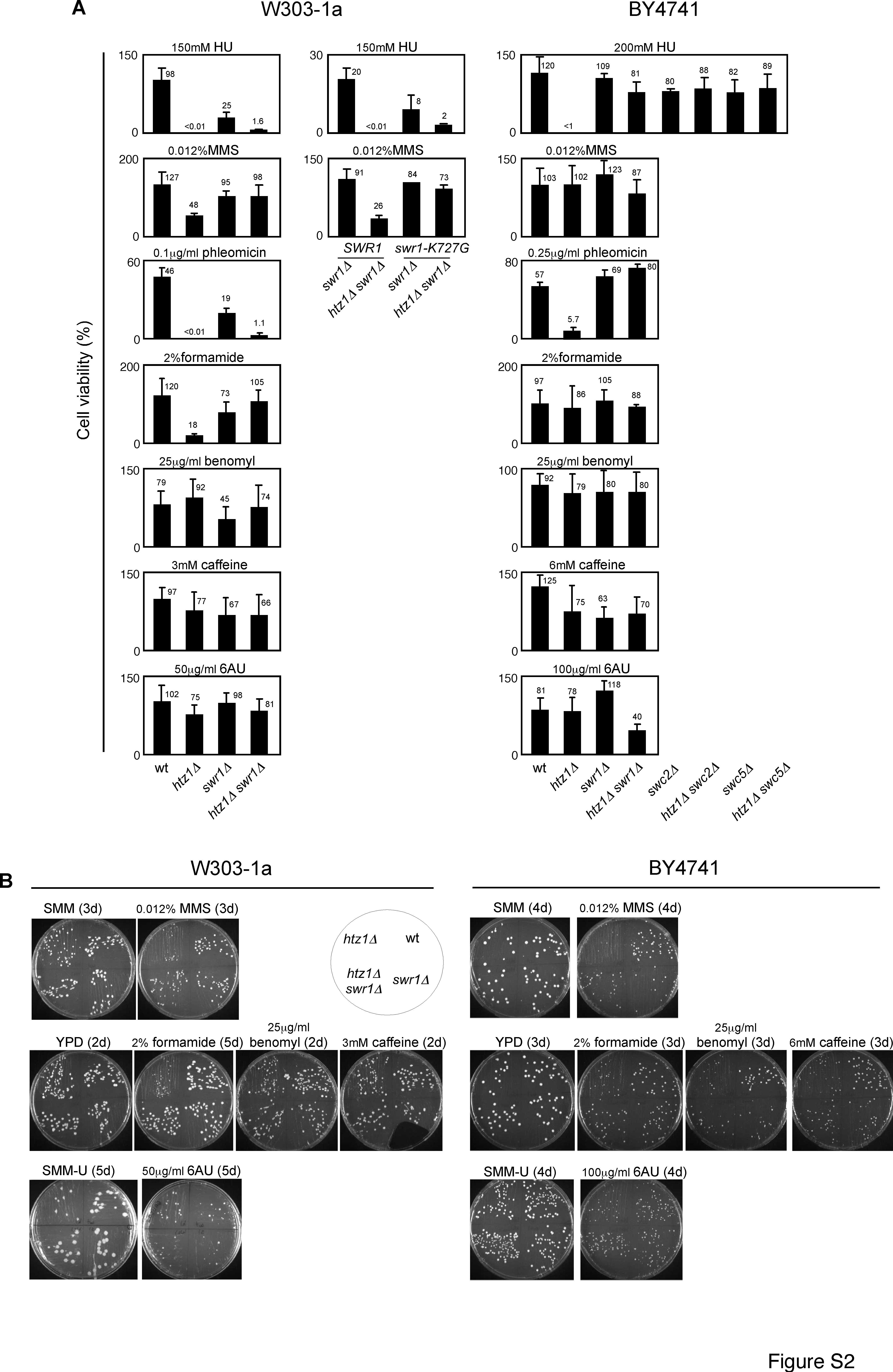

Supplement: Figure S2 — Cell viability (A) and growth (B) in response to DNA damage and stress conditions in W303-1a and BY4741 strains. (2.04 MB TIF) [file pone.0012143.s002.tif]

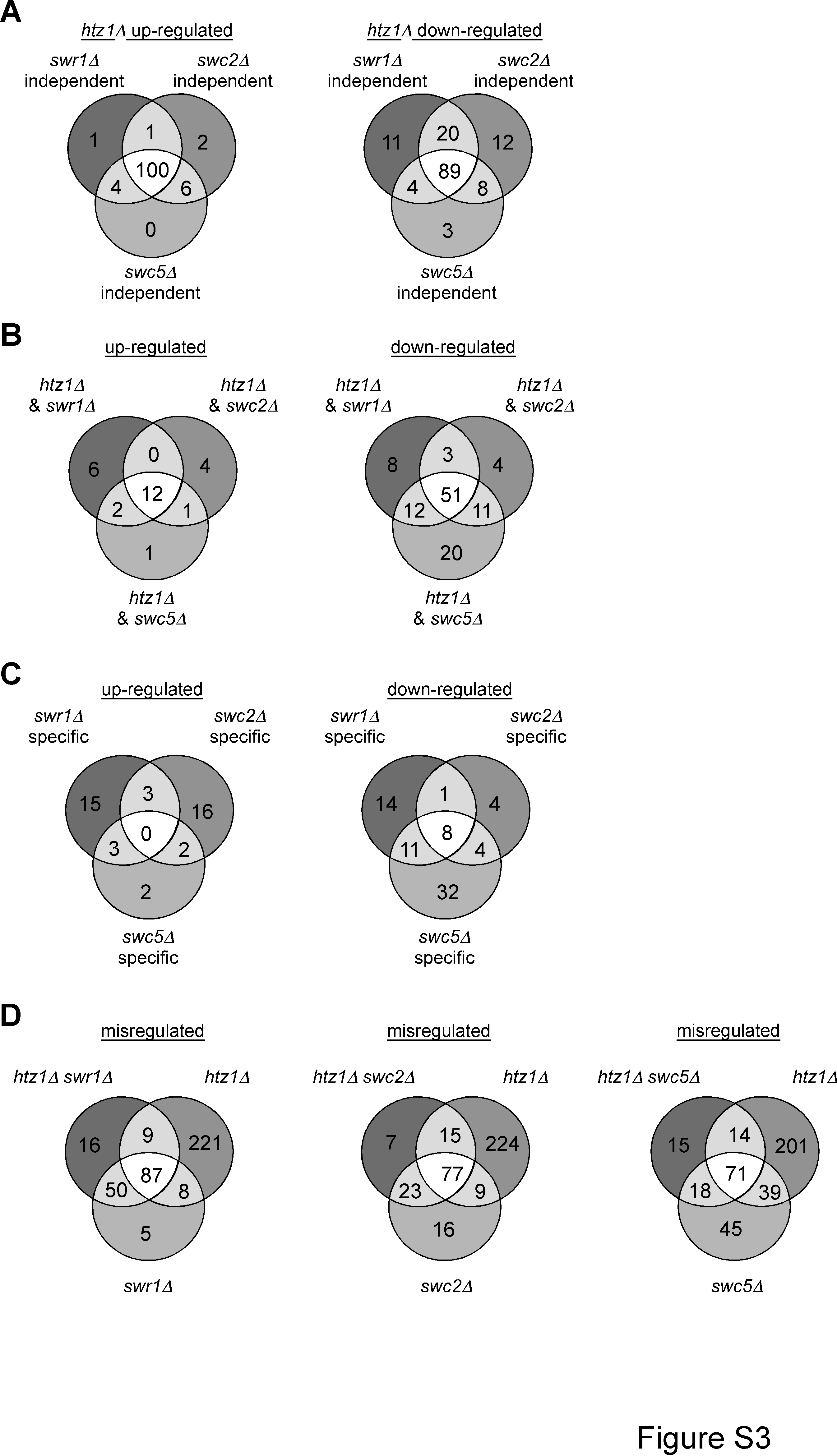

Supplement: Figure S3 — Venn diagrams showing the number of genes that were commonly misregulated (2-fold cutoff) by (A) (htz1Δ but no swr1Δ), (htz1Δ but no swc2Δ) and (htz1Δ but no swc5Δ) (B) (htz1Δ and swr1Δ), (htz1Δ and swc2Δ) and (htz1Δ and swc5Δ), (C) (swr1Δ but no htz1Δ), (swc2Δ but no htz1Δ) and (swc5Δ but no htz1Δ) and (D) (htz1Δ swr1Δ), (htz1Δ) and (swr1Δ); (htz1Δ swc5Δ), (htz1Δ) and (swc5Δ); (htz1Δ swc2Δ), (htz1Δ) and (swc2Δ). (0.19 MB TIF) [file pone.0012143.s003.tif]

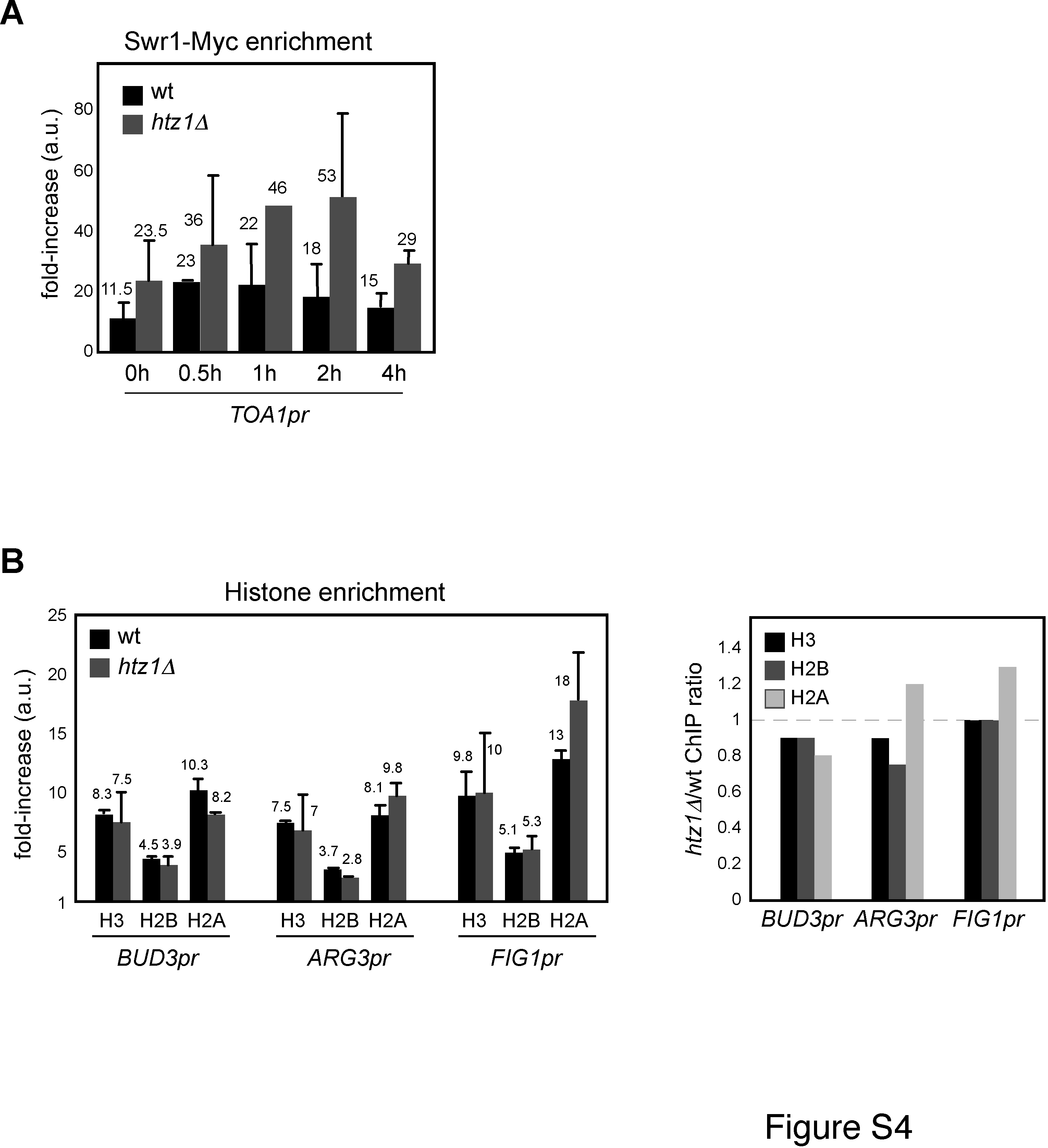

Supplement: Figure S4 — (A) Swr1-Myc enrichment at the TOA1 promoter as determined by ChIP analysis of samples in Figure 3C. Both I and IP DNA from cell extracts incubated either with anti-Myc antibody or IgG were amplified by real-time PCR (see Table S4 for oligos). The enrichment is graphed relative to the enrichment in the wild-type strain incubated with IgG, taken as 1. Similar results were obtained using as a control an untagged strain incubated with anti-Myc (data not shown). (B) Histone enrichment at the promoters of BUD3, ARG3 and FIG1 by ChIP analysis. Both I and IP DNA from cell extracts incubated either with anti-H3, anti-H2B, anti-H2A antibodies or IgG were amplified by real-time PCR with amplicons situated at the indicated regions (see Table S4 for oligos). The enrichment is graphed relative to the enrichment in the wild-type strain incubated with IgG, taken as 1. ChIP experiments were performed in BY4741 background. (0.15 MB TIF) [file pone.0012143.s004.tif]

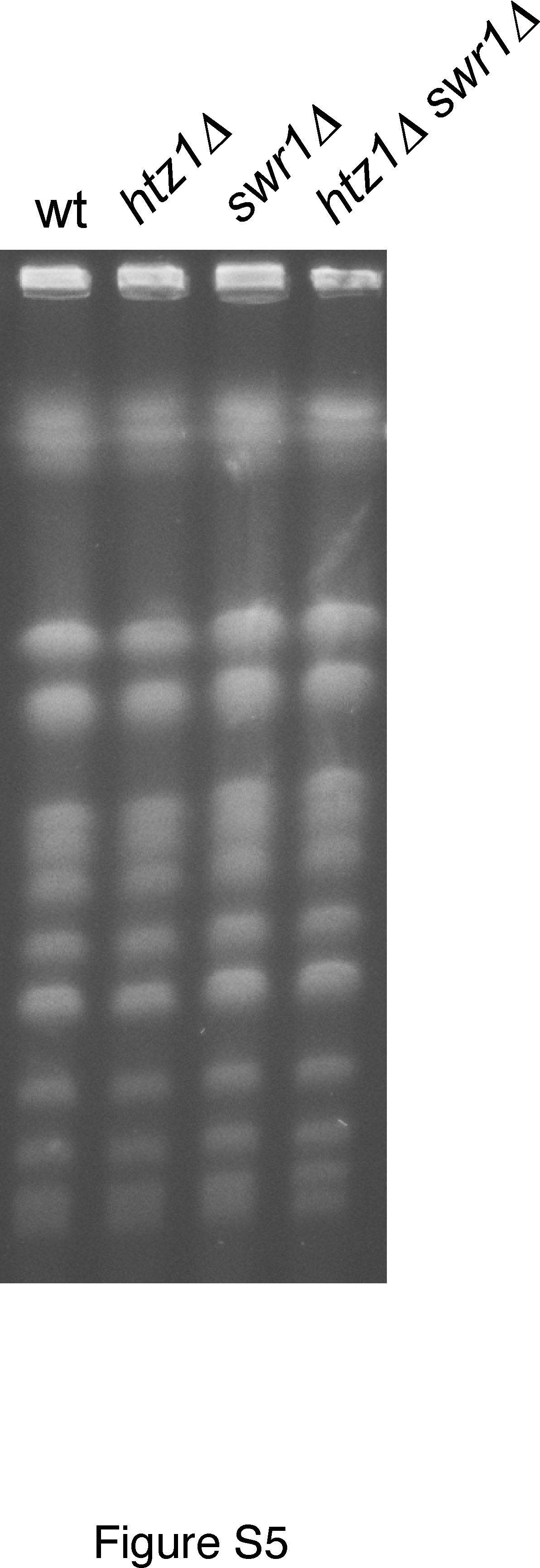

Supplement: Figure S5 — Analysis of spontaneous DNA breaks as determined by PFGE of yeast chromosomes in htz1Δ, swr1Δ, htz1Δ swr1Δ and wild type. (0.57 MB TIF) [file pone.0012143.s005.tif]
