# Supplementary material for: The SWR1 Histone Replacement Complex Causes Genetic Instability and Genome-Wide Transcription Misregulation in the Absence of H2A.Z
Source: PLoS One. 2010 Aug 12;5(8):e12143. doi: 10.1371/journal.pone.0012143 (PMC2920830; doi:10.1371/journal.pone.0012143)
Supplement: Table S1 — Transcription profiles of htz1Δ relative to swr1Δ, swc2Δ and swc5Δ. (0.27 MB DOC) [file pone.0012143.s006.doc]

***Table SI.*** *Transcription profiles of htz1∆ relative to swr1∆, swc2∆ and swc5∆*

| ***htz1∆ versus swr1∆*** | | | | | |
| --- | --- | --- | --- | --- | --- |
|  | **more than 2** | **genes** | **more than 1.5** | **genes** | | **more than 1.5** | **genes** | **more than 1.2** | **genes** | | --- | --- | --- | --- | | Up-regulated  by *htz1∆* | 311 | Sup. by *swr1∆* | 252 | | Una. by *swr1∆* | 49 | | Inc. by *swr1∆* | - | | Up-regulated  by *htz1∆*  and *swr1∆* | 84 | Sup. by *swr1∆* | 10 | | Una. by *swr1∆* | 74 | | Inc. by *swr1∆* | - | | Up-regulated  by *htz1∆*  and *swr1∆* | 84 | Sup. by *htz1∆* | - | | Una. by *htz1∆* | 84 | | Inc. by *htz1∆* | - | | Up-regulated  by *swr1∆* | 71 | Sup. by *htz1∆* | - | | Una. by *htz1∆* | 71 | | Inc. by *htz1∆* | - | | Down-regulated  by *htz1∆* | 355 | Sup. by *swr1∆* | 261 | | Una. by *swr1∆* | 93 | | Inc. by *swr1∆* | 1 | | Down -regulated  by *htz1∆*  and *swr1∆* | 197 | Sup. by *swr1∆* | 36 | | Una. by *swr1∆* | 148 | | Inc. by *swr1∆* | 13 | | Down -regulated  by *htz1∆*  and *swr1∆* | 197 | Sup. by *htz1∆* | - | | Una. by *htz1∆* | 197 | | Inc. by *htz1∆* | - | | Down -regulated  by *swr1∆* | 109 | Sup. by *htz1∆* | - | | Una. by *htz1∆* | 109 | | Inc. by *htz1∆* | - | |
| **Up-regulated**  **by *htz1∆*** | Up-regulated  by *htz1∆* | 106 | Sup. by *swr1∆* | 84 |  |
|  |  |  | Una. by *swr1∆* | 22 |  |
|  |  |  | Inc. by *swr1∆* | - |  |
|  | Up-regulated  by *htz1∆*  and *swr1∆* | 20 | Sup. by *swr1∆* | 2 |  |
|  |  |  | Una. by *swr1∆* | 17 |  |
|  |  |  | Inc. by *swr1∆* | 1 |  |
| **Up-regulated**  **by *swr1∆*** | Up-regulated  by *htz1∆*  and *swr1∆* | 20 | Sup. by *htz1∆* | - |  |
|  |  |  | Una. by *htz1∆* | 20 |  |
|  |  |  | Inc. by *htz1∆* | - |  |
|  | Up-regulated  by *swr1∆* | 21 | Sup. by *htz1∆* | - |  |
|  |  |  | Una. by *htz1∆* | 21 |  |
|  |  |  | Inc. by *htz1∆* | - |  |
| **down- regulated**  **by *htz1∆*** | Down-regulated  by *htz1∆* | 124 | Sup. by *swr1∆* | 90 |  |
|  |  |  | Una. by *swr1∆* | 34 |  |
|  |  |  | Inc. by *swr1∆* | - |  |
|  | Down -regulated  by *htz1∆*  and *swr1∆* | 74 | Sup. by *swr1∆* | 7 |  |
|  |  |  | Una. by *swr1∆* | 64 |  |
|  |  |  | Inc. by *swr1∆* | 3 |  |
| **down- regulated**  **by *swr1∆*** | Down -regulated  by *htz1∆*  and *swr1∆* | 74 | Sup. by *htz1∆* | - |  |
|  |  |  | Una. by *htz1∆* | 74 |  |
|  |  |  | Inc. by *htz1∆* | - |  |
|  | Down -regulated  by *swr1∆* | 34 | Sup. by *htz1∆* | - |  |
|  |  |  | Una. by *htz1∆* | 34 |  |
|  |  |  | Inc. by *htz1∆* | - |  |

| ***htz1∆ versus swc2∆*** | | | | | |
| --- | --- | --- | --- | --- | --- |
|  | **more than 2** | **genes** | **more than 1.5** | **genes** | | **more than 1.5** | **genes** | **more than 1.2** | **genes** | | --- | --- | --- | --- | | Up-regulated  by *htz1∆* | 323 | Sup. by *swc2∆* | 250 | | Una. by *swc2∆* | 73 | | Inc. by *swc2∆* | - | | Up-regulated  by *htz1∆*  and *swc2∆* | 72 | Sup. by *swc2∆* | 19 | | Una. by *swc2∆* | 52 | | Inc. by *swc2∆* | 1 | | Up-regulated  by *htz1∆*  and *swc2∆* | 72 | Sup. by *htz1∆* | - | | Una. by *htz1∆* | 72 | | Inc. by *htz1∆* | - | | Up-regulated  by *swc2∆* | 66 | Sup. by *htz1∆* | - | | Una. by *htz1∆* | 66 | | Inc. by *htz1∆* | - | | Down-regulated  by *htz1∆* | 370 | Sup. by *swc2∆* | 230 | | Una. by *swc2∆* | 102 | | Inc. by *swc2∆* | - | | Down -regulated  by *htz1∆*  and *swc2∆* | 182 | Sup. by *swc2∆* | 36 | | Una. by *swc2∆* | 142 | | Inc. by *swc2∆* | 4 | | Down -regulated  by *htz1∆*  and *swc2∆* | 182 | Sup. by *htz1∆* | - | | Una. by *htz1∆* | 182 | | Inc. by *htz1∆* | - | | Down -regulated  by *swc2∆* | 76 | Sup. by *htz1∆* | - | | Una. by *htz1∆* | 76 | | Inc. by *htz1∆* | - | |
| **Up-regulated**  **by *htz1∆*** | Up-regulated  by *htz1∆* | 109 | Sup. by *swc2∆* | 83 |  |
|  |  |  | Una. by *swc2∆* | 26 |  |
|  |  |  | Inc. by *swc2∆* | - |  |
|  | Up-regulated  by *htz1∆*  and *swc2∆* | 17 | Sup. by *swc2∆* | 1 |  |
|  |  |  | Una. by *swc2∆* | 16 |  |
|  |  |  | Inc. by *swc2∆* | - |  |
| **Up-regulated**  **by *swc2∆*** | Up-regulated  by *htz1∆*  and *swc2∆* | 17 | Sup. by *htz1∆* | - |  |
|  |  |  | Una. by *htz1∆* | 17 |  |
|  |  |  | Inc. by *htz1∆* | - |  |
|  | Up-regulated  by *swc2∆* | 21 | Sup. by *htz1∆* | - |  |
|  |  |  | Una. by *htz1∆* | 21 |  |
|  |  |  | Inc. by *htz1∆* | - |  |
| **down- regulated**  **by *htz1∆*** | Down-regulated  by *htz1∆* | 129 | Sup. by *swc2∆* | 77 |  |
|  |  |  | Una. by *swc2∆* | 52 |  |
|  |  |  | Inc. by *swc2∆* | - |  |
|  | Down -regulated  by *htz1∆*  and *swc2∆* | 69 | Sup. by *swc2∆* | 11 |  |
|  |  |  | Una. by *swc2∆* | 58 |  |
|  |  |  | Inc. by *swc2∆* | - |  |
| **down- regulated**  **by *swc2∆*** | Down -regulated  by *htz1∆*  and *swc2∆* | 69 | Sup. by *htz1∆* | - |  |
|  |  |  | Una. by *htz1∆* | 69 |  |
|  |  |  | Inc. by *htz1∆* | - |  |
|  | Down -regulated  by *swc2∆* | 17 | Sup. by *htz1∆* | - |  |
|  |  |  | Una. by *htz1∆* | 17 |  |
|  |  |  | Inc. by *htz1∆* | - |  |

| ***htz1∆ versus swc5∆*** | | | | | |
| --- | --- | --- | --- | --- | --- |
|  | **more than 2** | **genes** | **more than 1.5** | **genes** | | **more than 1.5** | **genes** | **more than 1.2** | **genes** | | --- | --- | --- | --- | | Up-regulated  by *htz1∆* | 336 | Sup. by *swc5∆* | 231 | | Una. by *swc5∆* | 102 | | Inc. by *swc5∆* | - | | Up-regulated  by *htz1∆*  and *swc5∆* | 62 | Sup. by *swc5∆* | 15 | | Una. by *swc5∆* | 47 | | Inc. by *swc5∆* | - | | Up-regulated  by *htz1∆*  and *swc5∆* | 62 | Sup. by *htz1∆* | 2 | | Una. by *htz1∆* | 60 | | Inc. by *htz1∆* | - | | Up-regulated  by *swc5∆* | 34 | Sup. by *htz1∆* | 2 | | Una. by *htz1∆* | 32 | | Inc. by *htz1∆* | - | | Down-regulated  by *htz1∆* | 333 | Sup. by *swc5∆* | 183 | | Una. by *swc5∆* | 150 | | Inc. by *swc5∆* | - | | Down -regulated  by *htz1∆*  and *swc5∆* | 219 | Sup. by *swc5∆* | 77 | | Una. by *swc5∆* | 142 | | Inc. by *swc5∆* | - | | Down -regulated  by *htz1∆*  and *swc5∆* | 219 | Sup. by *htz1∆* | 18 | | Una. by *htz1∆* | 201 | | Inc. by *htz1∆* | - | | Down -regulated  by *swc5∆* | 88 | Sup. by *htz1∆* | 20 | | Una. by *htz1∆* | 68 | | Inc. by *htz1∆* | - | |
| **Up-regulated**  **by *htz1∆*** | Up-regulated  by *htz1∆* | 110 | Sup. by *swc5∆* | 84 |  |
|  |  |  | Una. by *swc5∆* | 26 |  |
|  |  |  | Inc. by *swc5∆* | - |  |
|  | Up-regulated  by *htz1∆*  and *swc5∆* | 16 | Sup. by *swc5∆* | 4 |  |
|  |  |  | Una. by *swc5∆* | 12 |  |
|  |  |  | Inc. by *swc5∆* | - |  |
| **Up-regulated**  **by *swc5∆*** | Up-regulated  by *htz1∆*  and *swc5∆* | 16 | Sup. by *htz1∆* | 1 |  |
|  |  |  | Una. by *htz1∆* | 15 |  |
|  |  |  | Inc. by *htz1∆* | - |  |
|  | Up-regulated  by *swc5∆* | 7 | Sup. by *htz1∆* | 1 |  |
|  |  |  | Una. by *htz1∆* | 6 |  |
|  |  |  | Inc. by *htz1∆* | - |  |
| **down- regulated**  **by *htz1∆*** | Down-regulated  by *htz1∆* | 104 | Sup. by *swc5∆* | 63 |  |
|  |  |  | Una. by *swc5∆* | 41 |  |
|  |  |  | Inc. by *swc5∆* | - |  |
|  | Down -regulated  by *htz1∆*  and *swc5∆* | 94 | Sup. by *swc5∆* | 29 |  |
|  |  |  | Una. by *swc5∆* | 65 |  |
|  |  |  | Inc. by *swc5∆* | - |  |
| **down- regulated**  **by *swc5∆*** | Down -regulated  by *htz1∆*  and *swc5∆* | 94 | Sup. by *htz1∆* | 11 |  |
|  |  |  | Una. by *htz1∆* | 83 |  |
|  |  |  | Inc. by *htz1∆* | - |  |
|  | Down -regulated  by *swc5∆* | 55 | Sup. by *htz1∆* | 10 |  |
|  |  |  | Una. by *htz1∆* | 45 |  |
|  |  |  | Inc. by *htz1∆* | - |  |
