# Supplementary material for: The SWR1 Histone Replacement Complex Causes Genetic Instability and Genome-Wide Transcription Misregulation in the Absence of H2A.Z
Source: PLoS One. 2010 Aug 12;5(8):e12143. doi: 10.1371/journal.pone.0012143 (PMC2920830; doi:10.1371/journal.pone.0012143)
Supplement: Table S3 — Strains. (0.09 MB DOC) [file pone.0012143.s008.doc]

Table S3

| Strain^a^ | Relevant genotype | Source |
| --- | --- | --- |
| BY4741 | *MATa his3∆1 leu2∆0 ura3∆0 met15∆0* | Euroscarf |
| BYh1b1-6A | *MATa his3∆1 leu2∆0 ura3∆0 met15∆0 htz1∆::kanMX4 bar1Δ::hyg* | This work |
| Y03693 | *MATa his3∆1 leu2∆0 ura3∆0 met15∆0 swr1∆::kanMX4* | Euroscarf |
| BYh1s1-2A | *MATα his3∆1 leu2∆0 ura3∆0 met15∆0 htz1∆::kanMX4 swr1∆::kanMX4* | This work |
| BYh1s1-8D | *MATa his3∆1 leu2∆0 ura3∆0 met15∆0 htz1∆::kanMX4 swr1∆::kanMX4* | This work |
| Y04319 | *MATa his3∆1 leu2∆0 ura3∆0 met15∆0 swc2∆::kanMX4* | Euroscarf |
| BYh1s2-1B | *MATa his3∆1 leu2∆0 ura3∆0 met15∆0 htz1∆::kanMX4 swc2∆::kanMX4* | This work |
| BYh1s2-1C | *MATα his3∆1 leu2∆0 ura3∆0 lys2∆0 met15∆0 htz1∆::kanMX4 swc2∆::kanMX4* | This work |
| BYs1s2-1 | *MATa his3∆1 leu2∆0 ura3∆0 met15∆0 swc2∆::kanMX4 swr1Δ::hyg* | This work |
| BYs1s2-4 | *MATa his3∆1 leu2∆0 ura3∆0 met15∆0 swc2∆::kanMX4 swr1Δ::hyg* | This work |
| BYs1s2-5 | *MATa his3∆1 leu2∆0 ura3∆0 met15∆0 swc2∆::kanMX4 swr1Δ::hyg* | This work |
| BYh1s1s2-8 | *MATa his3∆1 leu2∆0 ura3∆0 met15∆0 htz1∆::kanMX4 swc2∆::kanMX4 swr1Δ::hyg* | This work |
| Y03371 | *MATa his3∆1 leu2∆0 ura3∆0 met15∆0 swc5∆::kanMX4* | Euroscarf |
| BYh1s5-4C | *MATa his3∆1 leu2∆0 ura3∆0 met15∆0 lys2∆0 htz1∆::kanMX4 swc5∆::kanMX4* | This work |
| BYh1s5-4D | *MATa his3∆1 leu2∆0 ura3∆0 met15∆0 htz1∆::kanMX4 swc5∆::kanMX4* | This work |
| BYh1s5-13B | *MATα his3∆1 leu2∆0 ura3∆0 met15∆0 htz1∆::kanMX4 swc5∆::kanMX4* | This work |
| BYh1s5-13D | *MATa his3∆1 leu2∆0 ura3∆0 met15∆0 htz1∆::kanMX4 swc5∆::kanMX4* | This work |
| SWR1TAP | *MATa his3∆1 leu2∆0 met15∆0 ura3∆0 SWR1TAP::HIS3MX6* | Open biosystems |
| SWR1TAPh1-1b | *MATα his3∆1 leu2∆0 met15∆0 ura3∆0 SWR1TAP::HIS3MX6 htz1∆::kanMX4* | This work |
| SWR1TAPs2-1c | *MATa his3∆1 leu2∆0 met15∆0 ura3∆0 SWR1TAP::HIS3MX6 swc2∆::kanMX4* | This work |
| SWR1TAPs5-7d | *MATa his3∆1 leu2∆0 met15∆0 ura3∆0 lys2∆0 SWR1TAP::HIS3MX6 swc5∆::kanMX4* | This work |
| HTZ1TAP | *MATa his3∆1 leu2∆0 met15∆0 ura3∆0 HTZ1TAP::HIS3MX6* | Open biosystems |
| HTZ1TAPs1-5C | *MATa his3∆1 leu2∆0 met15∆0 ura3∆0 HTZ1TAP::HIS3MX6 swr1∆::kanMX4* | This work |
| HTZ1TAPs5-2B | *MATa his3∆1 leu2∆0 met15∆0 ura3∆0 HTZ1TAP::HIS3MX6 swc5∆::kanMX4* | This work |
| W303-1aR5 | *MATa leu2-3,112 trp1-1 ura3-1 ade2-1 can1-100 his3-11 RAD5* | A. Aguilera |
| W303h1 | *MATa leu2-3,112 trp1-1 ura3-1 ade2-1 can1-100 his3-11 RAD5 htz1::hyg* | This work |
| W303s1 | *MATα leu2-3,112 trp1-1 ura3-1 ade2-1 can1-100 his3-11 RAD5 swr1::kanMX4* | This work |
| W303h1s1-1A | *MATa leu2-3,112 trp1-1 ura3-1 ade2-1 can1-100 his3-11 RAD5 htz1::hyg swr1::kanMX4* | This work |
| W303h1s1-3C | *MATα leu2-3,112 trp1-1 ura3-1 ade2-1 can1-100 his3-11 RAD5 htz1::hyg swr1::kanMX4* | This work |
| JDY22 | *MATα leu2-3,112 trp1-1 ura3-1 ade2-1 can1-100 his3-11 hta1-S129* hta2-S129** | Downs et al. 2000 |
| W303hta12sz-1C | *MATα leu2-3,112 trp1-1 ura3-1 ade2-1 can1-100 his3-11 hta1-S129* hta2-S129* htz1::hyg* | This work |
| JKM179 | *MATα hml::ADE1 hmr::ADE1 ade1-100 leu2-3,112 lys5 trp1::hisG ura3-S2 ade3::GAL::HO* | Lee et al. 1998 |
| JKMh1 | *MATα hml::ADE1 hmr::ADE1 ade1-100 leu2-3,112 lys5 trp1::hisG ura3-S2 ade3::GAL::HO htz1::hyg* | This work |
| JKMs1 | *MATα hml::ADE1 hmr::ADE1 ade1-100 leu2-3,112 lys5 trp1::hisG ura3-S2 ade3::GAL::HO swr1::KanMx4* | This work |
| JKMh1s1 | *MATα hml::ADE1 hmr::ADE1 ade1-100 leu2-3,112 lys5 trp1::hisG ura3-S2 ade3::GAL::HO htz1::hyg swr1::KanMx4* | This work |
| JKM-MycS | *MATα hml::ADE1 hmr::ADE1 ade1-100 leu2-3,112 lys5 trp1::hisG ura3-S2 ade3::GAL::HO SWR1MYC::KanMx4* | This work |
| JKMh1-MycS | *MATα hml::ADE1 hmr::ADE1 ade1-100 leu2-3,112 lys5 trp1::hisG ura3-S2 ade3::GAL::HO htz1::hyg SWR1MYC::KanMx4* | This work |
| BY4733 | *MATa his3-200 leu2 met15 trp1-63 ura3* | Research Genetics |
| GA-2264 | *MATa his3-200 leu2 met15 trp1-63 ura3* *ino80∆TRP1* | Shen et al. 2000 |
| BY33h1 | *MATa his3-200 leu2 met15 trp1-63 ura3* *htz1::hyg* | This work |
| BY33i80h1 | *MATa his3-200 leu2 met15 trp1-63 ura3* *ino80∆TRP1htz1::hyg* | This work |
| BY33s1 | *MATa his3-200 leu2 met15 trp1-63 ura3 swr1::hyg* | This work |
| BY33i80s1 | *MATa his3-200 leu2 met15 trp1-63 ura3* *ino80∆TRP1swr1::hyg* | This work |
| BY33h1s1 | *MATa his3-200 leu2 met15 trp1-63 ura3 swr1::hyg* *htz1::kanMX4* | This work |

^a^All strains are isogenic to BY4741, except W303 and JDY22 strains that are isogenic to W303-1a, BY33 and GA-264 that are isogenic to BY4733, and JKM strains that are isogenic to JKM179.
