# Supplementary material for: The SWR1 Histone Replacement Complex Causes Genetic Instability and Genome-Wide Transcription Misregulation in the Absence of H2A.Z
Source: PLoS One. 2010 Aug 12;5(8):e12143. doi: 10.1371/journal.pone.0012143 (PMC2920830; doi:10.1371/journal.pone.0012143)
Supplement: Table S4 — Oligos. (0.05 MB DOC) [file pone.0012143.s009.doc]

**Table S4**

| oligo^a^ | Sequence |
| --- | --- |
| ForBUD3prom | AACACTAGTTTGTCTGTACTCGCATTC |
| RevBUD3prom | ACAGCCCGAACACGTAATGAG |
| ForBUD3 | CTGCGTCTTCTTATCCTGAAAAACT |
| RevBUD3 | ATTCGTCGCCTTCCCAATTA |
| ForTOA1 | GAAAAGATGAAGAAGCCAAATAACAA |
| RevTOA1 | TGAGCTTGCCCCCGATAAT |
| ForYNL116Wp | AGAAGTCGAGAAAGTATAGCAAC |
| RevYNL116Wp | TTCCACGGTATATCCTTTGCTGA |
| ForSSM4p | GAGCCCCTACAGGGCTATCT |
| RevSSM4p | CTCAATCGATGCGTTTTCAA |
| ForHOcs | ACAAAATGCAGCACGGAATA |
| RevHOcs | TCTTCCCAATATCCGTCACC |
| ForP1kbL | GGGTTCTCGATGTTCTTTGC |
| RevP1kbL | AAAATAAATCGGCGGATGG |
| ForP0.6kbR | TGGCGGAGGTTGTTTATCTT |
| RevP0.6kbR | AAGACGATGGGGAGTTTCAA |
| ForFIG1 5’ | ATGCCCAGAATATTTGCATTAGC |
| RevFIG1 5’ | GACTGATTTGACGGGTTGTAACAG |
| ForARG3prom | ATATAAATCGACTTTTCACCTCTA |
| RevARG3prom | GGTAACGAGTGTACAAGTTGATGGG |

^a^ Oligos used for real-time PCR amplifications
